# Revealing connectivity patterns of deep brain stimulation efficacy in Parkinson’s disease

**DOI:** 10.1038/s41598-024-80630-9

**Published:** 2024-12-30

**Authors:** Eva Výtvarová, Martin Lamoš, Jaroslav Hlinka, Sabina Goldemundová, Ivan Rektor, Martina Bočková

**Affiliations:** 1 Brain and Mind Research Program, Central European Institute of Technology (CEITEC), Masaryk University, Brno, Czech Republic; 2 Faculty of Informatics, Masaryk University, Brno, Czech Republic; 3 Department of Complex Systems, Institute of Computer Science, Czech Academy of Sciences, Prague, Czech Republic; 4 National Institute of Mental Health, Klecany, Czech Republic; 5 First Department of Neurology, Masaryk University School of Medicine, St. Anne’s Hospital, Brno, Czech Republic

**Keywords:** Functional connectivity, EEG, Connectivity patterns, Subthalamic nucleus, Parkinson’s disease, Deep brain stimulation, Neuroscience, Biomarkers, Neurology

## Abstract

The aim of this work was to study the effect of deep brain stimulation of the subthalamic nucleus (STN-DBS) on the subnetwork of subcortical and cortical motor regions and on the whole brain connectivity using the functional connectivity analysis in Parkinson’s disease (PD). The high-density source space EEG was acquired and analyzed in 43 PD subjects in DBS on and DBS off stimulation states (off medication) during a cognitive-motor task. Increased high gamma band (50–100 Hz) connectivity within subcortical regions and between subcortical and cortical motor regions was significantly associated with the Movement Disorders Society – Unified Parkinson’s Disease Rating Scale (MDS-UPDRS) III improvement after DBS. Whole brain neural correlates of cognitive performance were also detected in the high gamma (50–100 Hz) band. A whole brain multifrequency connectivity profile was found to classify optimal and suboptimal responders to DBS with a positive predictive value of 0.77, negative predictive value of 0.55, specificity of 0.73, and sensitivity of 0.60. Specific connectivity patterns related to PD, motor symptoms improvement after DBS, and therapy responsiveness predictive connectivity profiles were uncovered.

**Supplementary Information:**

The online version contains supplementary material available at 10.1038/s41598-024-80630-9.

## Introduction

Deep brain stimulation (DBS) is a well-established neuromodulation therapy that has been used in routine clinical practice to treat symptoms in various neurological and psychiatric disorders. Advanced motor symptoms of Parkinson’s disease (PD) are the most frequent indication for DBS, and here, it represents the second most crucial therapeutic progress after the introduction of levodopa^1–4^. The clinical use of DBS is among the most important advances in clinical neurosciences in the past two decades- for review, see^5^. Generally, it is a very successful therapy, but still, there are some limitations, including insufficient clinical response and occasionally adverse side effects^6–8^. Recent research focuses on electrophysiological studies that dynamically push the approaches toward treatment optimization and individual strategies. Adaptive DBS (aDBS) based on intracranial analysis of local field potentials represents the primary current progress^9–11^. Beta power hypersynchrony (13–35 Hz) in motor circuits is the main pathophysiological marker correlating to the severity of hypokinesia and rigidity and is known to be suppressible by dopaminergic medication as well as by DBS^12–16^. However, many other oscillatory changes have been described in relation to PD motor symptoms, including disturbed gamma power and high-frequency oscillations and their cross-frequency interactions. Mainly, gamma is considered to have a prokinetic significance^17–19^.

More and more attention is also being focused on surface EEG analysis, potentially revealing other clinically valuable biomarkers for improving DBS clinical efficacy and refining the pre-implantation criteria (for review, see^20^). PD is linked to various electrophysiological signs, from changes in EEG-detected background activity to complex functional connectivity disturbances on the cortico-subcortical and cortico-cortical levels (for review, see^21^). Advanced acquisition systems and novel analytical methods such as network connectivity measures, automated classifiers, and machine learning approaches offer significant promise for future clinical applications. For instance, some studies predicting DBS outcomes have already been published^22–25^. In this work, we have used connectivity pattern analysis of high-density EEG (HDEEG) to evaluate and explain the clinical DBS effect in PD on the structures involved in motor control and important in the context of PD pathophysiology and on the whole brain level.

In contrast to the majority of earlier studies, we used task-related HDEEG data during movement performance with a cognitive load for the analysis. We believe that the DBS effect is better reflected in such data than in a resting state condition as the motor and cognitive task performance mirrors basic activities of daily living that are impaired in PD. The main aims of this study were to identify differences between good and suboptimal responders and to identify potential connectivity patterns with a predictive value for DBS therapy outcomes that could be helpful in future clinical practice.

## Methods

### Patients and experimental protocol

The experimental protocol and processing of EEG data relate to our study targeting functional network topology differences in subjects with various reaction times in the motor cognitive paradigm^26^. High-density scalp EEG was recorded in the shielded cabin in 43 PD patients (age 61.07 ± 6.76; 14 females, 29 males) treated by STN-DBS with the use of an HDEEG Electrical Geodesics, Inc. (EGI GES 400 MR) system with 256 channels, 1 kHz sampling rate, and Cz electrode as a reference. The subjects performed a visual oddball three-stimuli experiment^27,28^ both in DBS ON and OFF conditions – 17 patients first with DBS off, then turned on, 26 patients first with DBS turned on and then turned off. The change of the order of the conditions in approximately half of our patients was intended in order to exclude the effect of learning and the other possible psychological phenomena that could influence the results (for example^29^) in the repeated task with a cognitive load. Between both recordings, there was a break of 15 min (a longer interval would be more appropriate to exclude the duration of the DBS effect, but we had to respect the patient´s safety and comfort, as they were in the OFF medication/OFF stimulation state). The experimental protocol is shown in Table 1. The frequent (non-target, standard) stimuli, which were 70% of all the stimuli, were small blue circles. These were not to be followed by any reaction. The target stimuli, which were 15% of all the stimuli, were larger blue circles, and the patients had to press a response button (by the dominant right hand) at the time of the target detection. The distractors (rare non-target stimuli), which were 15% of the stimuli, were black and white checkerboards; no response was required. The task lasted 14 min, with each stimulus presented for 200ms in random order and 4s interstimulus intervals. Patients were in the OFF-medication state, with their medication withdrawn for 12 h to exclude the effect of the dopaminergic therapy (dopamine agonists were withdrawn for 24 h. We are aware that the effect of dopamine agonists can last up to 72 h, but it is not acceptable to reduce the medication for such a long period for research reasons).

**Table 1:** Experimental protocol.

Stimulus	Description	Image	Response	Trials	Proportion
Target	Large blue circle		Press a button	30	15%
Non-target (frequent)	Small blue circle		No response	140	70%
Distractor	Black & white checkerboard		No response	30	15%

Legend: Randomized order of stimulus types, each stimulus lasting 200ms, with interstimulus interval 4s of blank screen.

Patients’ current clinical condition and the severity of motor symptoms were measured by the Movement Disorders Society – Unified Parkinson’s Disease Rating Scale (MDS-UPDRS) III, both in DBS ON and OFF states. They underwent subsequent examinations in DBS ON condition comprising these neuropsychological tests: Digit span, word list 1 – immediate verbal memory, word list 2 – immediate verbal memory (Wechsler Memory Scale-III), Stroop test interference, and Mattis total score. Patients did not express signs of dementia (Mattis cut-off score for dementia was ≤ 137) or major depression and did not have any other severe psychiatric symptoms (impulse control disorders, hallucinations, psychosis). We compared preoperative and postoperative Mattis total scores, where available, and there were no significant differences found (*N* = 25 subjects; paired t-test, *p* = 0.9650). Patients’ characteristics are summarized in Supplementary Tables S1 and S2. The study was approved by the local ethics committee (Ethics Board of the Faculty of Medicine, Masaryk University, Brno, Czech Republic), and patients were informed about the study and gave their informed consent. The study was carried out in accordance with relevant guidelines and regulations.

### DBS setting and EEG processing

Lead-DBS software (www.lead-dbs.org^30^) was used to verify the optimal DBS lead positions. Preoperative MRI T1 images were co-registered to postoperative CT with DBS leads, all normalized into the MNI space and overlaid with DISTAL atlas^31^. All patients who participated in the study did so after a full course of clinical DBS optimization- clinical setting based on the best effect on motor symptoms without adverse effects, stable for at least three months (see Table S1 in the supplementary material for patients’ DBS settings).

**Fig. 1:**
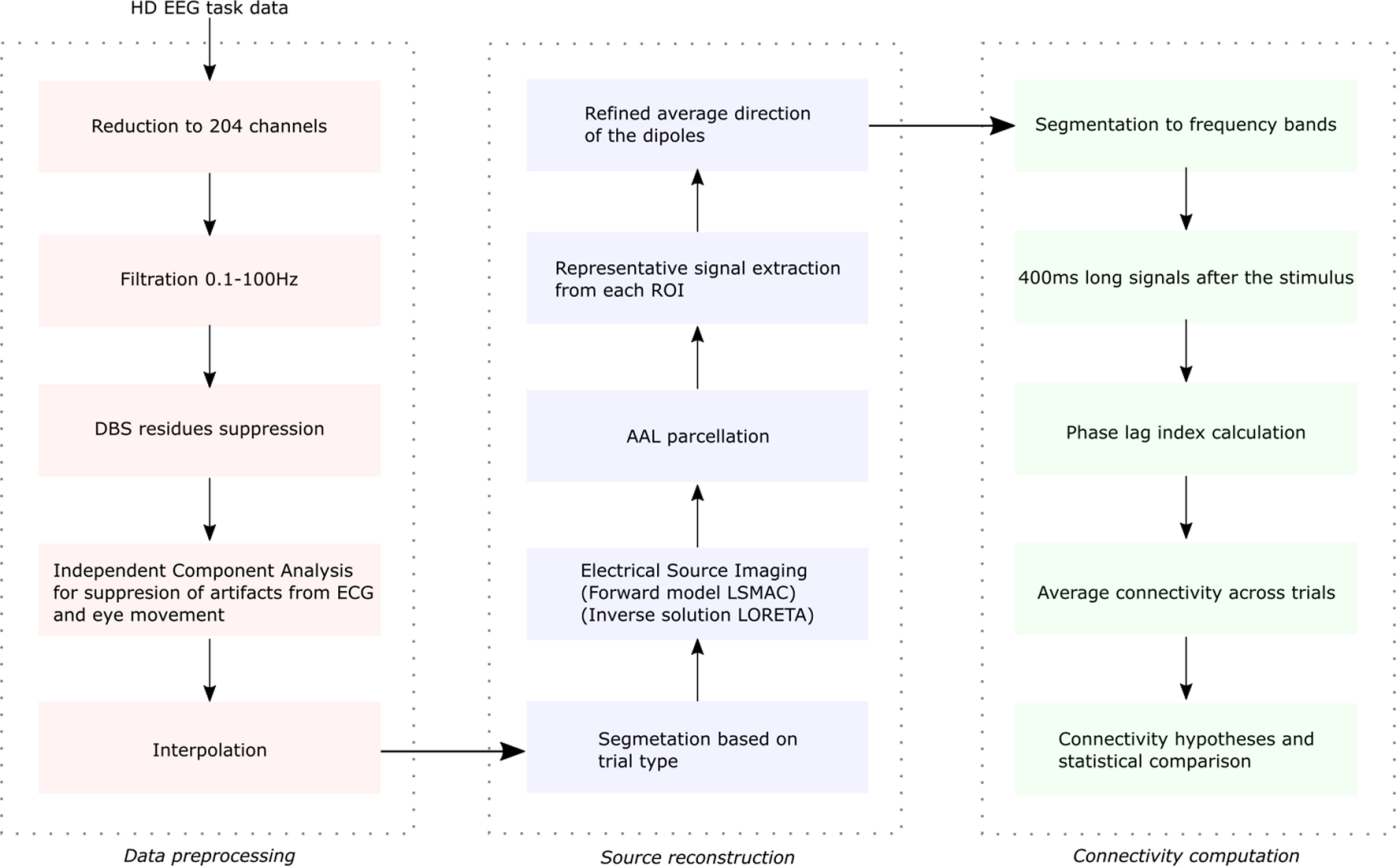
Diagram of individual EEG preprocessing steps.

Data preprocessing is summarized in Fig. 1. Functions from EEGLAB toolbox^32^ running under MATLAB 2014b were used to preprocess the EEG data. Facial and neck-line electrodes were discarded since they are frequently contaminated with muscle artifacts^33^, leaving the same 204 channels in each patient for analysis. Using a second-order Butterworth filter, 12dB/octave roll-off, and forward and backward passes to eliminate phase shifts^34^, the data were filtered to 0.1–100 Hz bandwidth.

After visual inspection (using SignalPlant software^35^) of the EEG in the spectral domain, the residuals of DBS artifact–aliased frequencies (narrow peaks with substantially higher magnitude than background activity) under 100 Hz were filtered out using a Fast Fourier Transform (FFT) filter (zeroing spectral lines on frequencies contaminated by DBS artifact residues^26,36,37^). An example of the time domain and power spectrum of the raw and filtered signal is visualized in Supplementary Figure S1. This approach was also used in other studies^26,37,38^. No more than five frequency peaks were suppressed in each recording. DBS artifact filtration was performed in the DBS ON recording and replicated in the same subject’s DBS OFF recording to keep the same pipeline for both conditions.

Artifacts related to eye movements and ECG were suppressed by Independent Component Analysis (ICA). Since it is unlikely for an automated procedure to effectively separate artifacts without any form of supervision^39^, manually selected independent components corresponding to artifacts were discarded, and data were back-reconstructed without these components. A maximum of four components were discarded in any recording.

Targeting our experimental paradigm, each continuous EEG signal was fragmented into 3s-long trials around stimuli ranging from 1s before to 2s after the stimulus. Trials containing artifacts not suppressed by the preprocessing pipeline (e.g., muscles and electrode “pop”) were excluded from the following analyses. A minimum number of target trials were controlled; no subject had fewer than 20 target trials. Retained trials were re-referenced to the average reference. They underwent a source reconstruction procedure: electrical source imaging using Cartool^40^ and MATLAB 2014b were used. The forward LSMAC model was constructed using the MNI template, followed by the LORETA algorithm for inversion. The grid of 5000 solution points in the grey matter was parcellated based on the AAL atlas^41^. Centroids from 90 ROIs, excluding vermis and cerebellum, were then projected to a refined average orientation^42^.

The ROI activity time series around each stimulus was then filtered into commonly used EEG frequency bands: delta 0.1–4 Hz, theta 4–8 Hz, alpha 8–12 Hz, beta 12–30 Hz, low gamma 30–50 Hz, and high gamma 50–100 Hz. The Butterworth filter of second order, 12dB/octave roll-off, and forward and backward passes were used, similarly as in^34^. The range of the high gamma band was chosen to cover synchronization activity, as observed by^43,44^. A published work^45^ has provided direct evidence that scalp EEG data can detect signals from subcortical structures, such as the thalamus and the basal ganglia, which are crucial in the pathophysiology of PD. Our electrical source imaging pipeline is very similar to that study, and we believe that we were able to reconstruct meaningful signals from these deep regions of the brain.

A 400ms long fraction of the reconstructed time series from 200ms to 600ms after the stimulus was used for connectivity metric calculation. The time interval was selected to avoid the immediate sensory reaction to the visual stimuli while capturing motor cognitive processing (see examples of evoked responses and time-frequency analysis in the Supplementary materials Figures S8 and S9)^27^. Phase-lag indices (PLI) (using code from https://github.com/NBT-Analytics/NBTpublic/issues/3 ) between all ROIs were computed, thus creating functional connectivity (FC) matrices of synchronization across the whole brain, except the vermis and cerebellum, for each subject, DBS condition, frequency band, and trial. The PLI was chosen to avoid volume conduction; other metrics, such as coherence and phase-amplitude coupling, are sensitive to signal amplitude changes, which can create type 1 errors^46,47^. To increase individual signal-to-noise ratios (SNRs) for further analyses, we averaged each subject’s FC matrices across trials for each DBS condition and frequency band. In this work, we concentrate only on analyzing the target stimuli of the oddball task, as we are focused on motor-cognitive task performance that corresponds best, in our opinion, to the daily activities involving both movement and executive processing that are impaired in patients with PD.

### Data analysis

#### Differences in motor subnetwork FC between DBS ON and OFF states

Since DBS primarily improves motor symptoms, a bilateral motor subnetwork of seven AAL ROIs was extracted. The motor subnetwork comprises these regions of basal ganglia-thalamo-cortical circuits: Precentral L + R, Supplementary Motor Area (SMA) L + R, Postcentral L + R, Caudate L + R, Putamen L + R, Pallidum L + R, and Thalamus L + R, which were chosen based on our knowledge as these structures are involved in motor control and are important in the context of PD pathophysiology. Unfortunately, STN could not be included, as the area of STN is too small and is not within the reach of scalp recording, even with the use of the source reconstruction approach, where the grid of solution points has 6 mm resolution and template MRI scan is used for the forward modeling^48,49^. The representative within-motor-subnetwork connectivity was computed for each subject, frequency band, and DBS condition as the first eigenvariate of the connections within the motor subnetwork. The Wilcoxon sign rank test was used to evaluate the differences caused by DBS due to non-gaussian distribution in some of the frequency bands, *p* < 0.05, False Discovery Rate (FDR) corrected, with age and gender as covariates. To capture shifts in the importance of individual motor regions caused by DBS in this subnetwork, each region’s average (seed) connectivity with the rest of the motor subnetwork was computed for each subject and DBS condition in significant frequency bands. They were evaluated by the Wilcoxon sign rank test, *p* < 0.05, corrected for multiple comparisons by FDR.

#### The DBS OFF motor subnetwork FC and motor symptoms improvement by DBS

An analysis focusing on the connectivity within the motor subnetwork and its relevance to the efficacy of DBS was carried out with the aim of linking results from the previous section to clinical observations. MDS-UPDRS III quantified motor symptoms in ON and OFF conditions, and its improvement was quantified as MDS-UPDRS_diff_ = MDS-UPDRS_ON_ − MDS-UPDRS_OFF_. The DBS OFF within-motor subnetwork FC, significantly altered by DBS, was correlated with MDS-UPDRS_diff_ using Spearman’s correlation with age and gender as covariates, *p* < 0.05, FDR corrected.

#### Whole brain differences in connectivity between DBS ON and OFF states

The motor subnetwork analyses were followed by the whole brain analyses, which aimed to exploratorily reveal other than motor subnetwork-related effects of DBS. The differences at the connection level in whole brain connectivity between DBS ON and DBS OFF conditions were evaluated. A Wilcoxon sign rank test for *p* < 0.05, FDR corrected, was used.

#### The DBS ON connectivity patterns and cognition

The relationship between connectivity and cognition was investigated. To reduce the dimensionality of the whole brain FC, the Principal Component Analysis (PCA) was applied on PLI matrices in the DBS ON state across patients for each frequency band to extract frequency-specific whole-brain connectivity patterns (CP_ON_s). The first few components in descending order of eigenvalues were selected/utilized for the analysis. The number of CP_ON_s was chosen from variances explained by components for each frequency band separately by an elbow method (Sebastien De Landtsheer (2024). kmeans_opt (https://www.mathworks.com/matlabcentral/fileexchange/65823-kmeans_opt), MATLAB Central File Exchange. Retrieved March 6, 2024. The method was repeated 100 times, and the most frequent result was used as an indicator of an elbow). Cognition was quantified by T-scores of neuropsychological tests (Digit span, word list 1, word list 2, Stroop test interference, and Mattis total score). Spearman’s partial correlations were then used with age and gender as covariates, *p* < 0.05, FDR corrected, to quantify relationships between CP_ON_s’ scores and T-scores of tests. The plots of explained variances are shown in Supplementary Figure S3.

#### The DBS OFF connectivity profile of optimal responders – classification analysis

The last aim of this study was to detect a possible connectivity profile that would differentiate good responders to DBS (optimal) from those who don’t show the expected level of improvement. This profile could be used to predict the clinical response to DBS from the DBS OFF connectivity before surgery. The responsiveness to DBS was quantified by the difference in motor symptoms measured in ON and OFF states quantified by MDS-UPDRS_diff_.

**Fig. 2:**
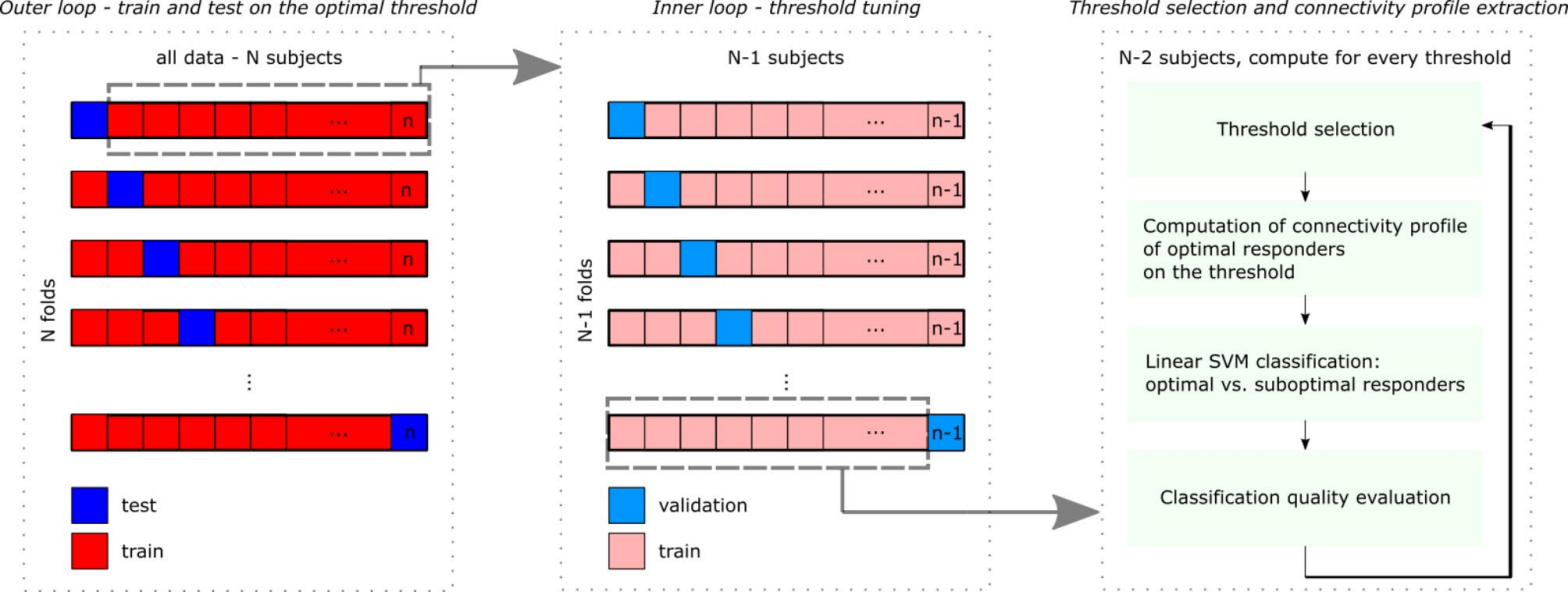
The scheme of nested leave-one-out cross-validation. Figure legend: The procedure was repeated 100 times, and the average PPV, NPV, specificity, and sensitivity across repetitions were computed for the optimal response threshold.

The whole procedure is depicted in Fig. 2. The nested leave-one-out cross-validation design consisting of an inner and outer loop was adopted. In the inner loop, a threshold for optimal responders was tuned, while the outer loop validated the classifier on an independent sample. A linear Support Vector Machine (SVM) classifier was used, and its quality was assessed by the positive predictive value (PPV), negative predictive value (NPV), sensitivity, and specificity, whose values range from zero (poor classification) to one (excellent classification). The PPV reflects the proportion of subjects marked as optimal who truly are the optimal responders to DBS. The NPV refers to how likely it is for a subject to truly be a suboptimal responder to DBS in cases of him/her being classified as suboptimal. The sensitivity is the probability of a truly optimal responder to DBS being marked by the classifier as optimal. And the specificity is the probability of a poor responder to DBS being marked as suboptimal.

In the outer loop, one subject was removed as a test subject, and the rest went into the inner loop. There, another subject was eliminated. On the remaining validation dataset, the UPDRS_diff_s were computed for each subject and sorted. They range from − 9 (poor improvement of motor symptoms after DBS) to -41 (the best-measured improvement). A sweep across different levels of UPDRS_diff_ (= response threshold for identifying optimal responders) was undertaken. Each response threshold level identified a subset of subjects as possibly-optimal, and the average difference in DBS ON and DBS OFF connectivity was computed for this subset. The values of connectivity differences, excluding connections that weren’t affected enough (less than four standard deviations from the average value in each frequency band), were used to define a characteristic connectivity profile of the DBS effect of these possibly-optimal responders. This process is depicted in the scheme in Supplementary Figure S5. The identified profile was used to extract DBS OFF values of the profile’s connections and input them into the classifier.

The classification was carried out for every UPDRS_diff_ level (response threshold). The best ratio between the PPV, NPV, specificity, and sensitivity in the inner loop specified the most discriminable response threshold. The most frequent response threshold across N folds of the outer loop then identified the resulting connectivity profile. A hundred repetitions of the procedure were run to provide reliable results. A detailed description of the classification procedure is reported in the Supplementary Material.

## Results

### Differences in motor subnetwork FC between DBS ON and OFF states

A significant (*p* < 0.001, FDR corrected) increase in the within-motor-subnetwork connectivity was observed in the DBS ON compared to DBS OFF in the high gamma band (50–100 Hz). Posthoc testing of average connectivity of individual motor regions in the high gamma band identified significantly (*p* < 0.05, FDR corrected) increased connectivity of Precentral L (*p* = 0.007), Precentral R (*p* = 0.006), SMA L (*p* < 0.001), Postcentral L (*p* = 0.005), Caudate L (*p* = 0.020), Putamen L (*p* = 0.007), and Pallidum L (*p* = 0.025) in DBS ON as compared to DBS OFF state. The same results, except increased connectivity of Pallidum L, were found significant (*p* < 0.05, FDR corrected) when correcting for the effects of age, gender, PD duration, DBS duration, PD severity, and levodopa equivalent dose (LED). The average motor subnetworks for DBS OFF and DBS ON states are visualized in Supplementary Figure S2.

### The DBS OFF motor subnetwork FC and motor symptoms improvement by DBS

**Fig. 3:**
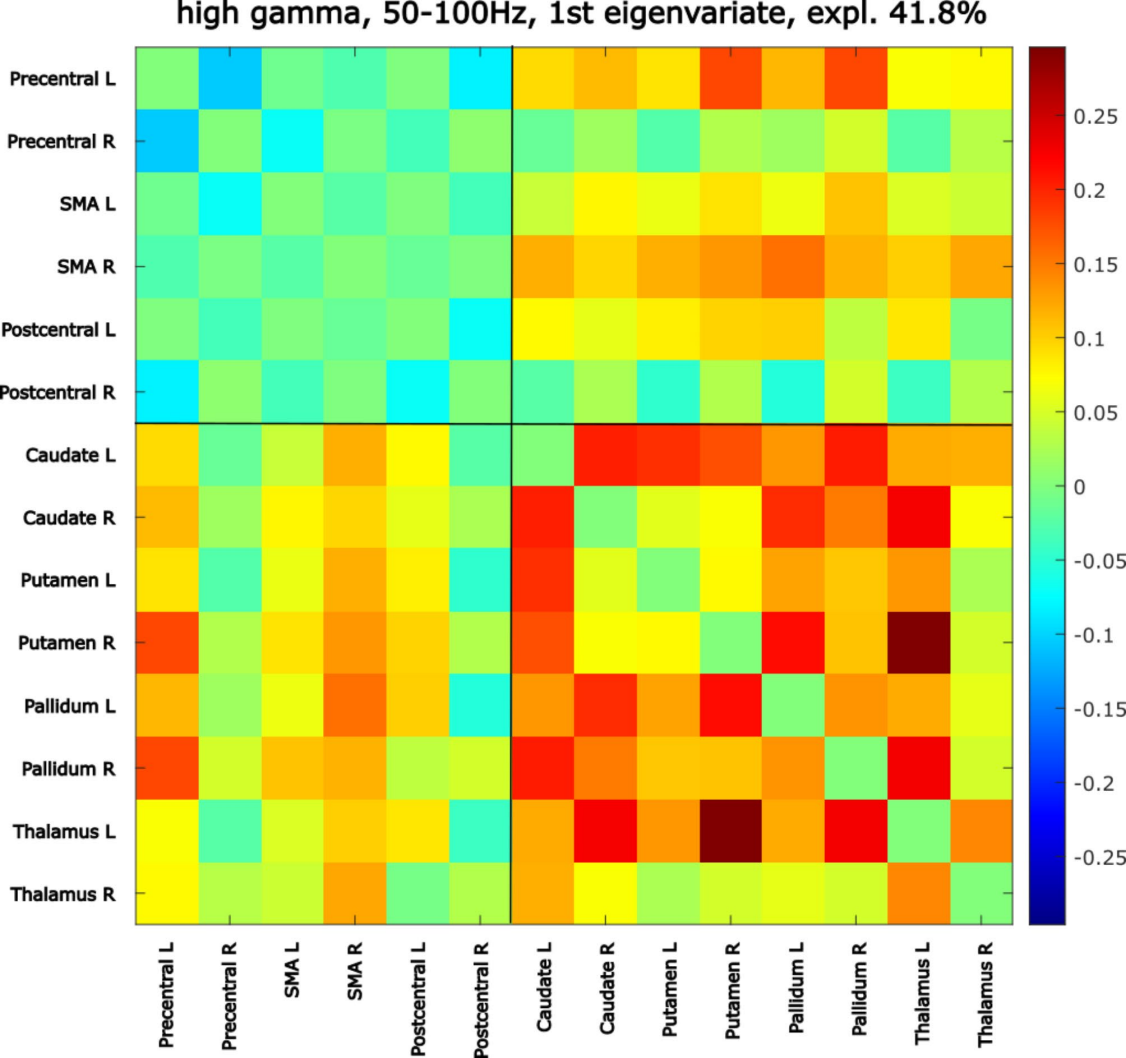
DBS OFF motor subnetwork pattern related to motor symptoms improvement. Figure legend: High gamma band 50–100 Hz, explains 41.8% variability. Correlation with MDS-UPDRS_diff_. ρ=-0.36, *p* = 0.021, FDR corrected. ROIs are sorted in this order: cortical ROIs (Precentral L, Precentral R, SMA L, SMA R, Postcentral L, Postcentral R), subcortical ROIs (Caudate L, Caudate R, Putamen L, Putamen R, Pallidum L, Pallidum R, Thalamus L, Thalamus R). Color-coded values correspond to principal component coefficients (PCA loadings): red: high PCA loadings, blue: low PCA loadings.

To relate the high gamma motor subnetwork FC found increased in DBS ON compared to DBS OFF in the previous step, with the improvement of motor symptoms thanks to DBS, we correlated the within-motor subnetwork FC in DBS OFF with MDS-UPDRS_diff_. We observe a significant correlation (ρ=-0.36, *p* = 0.021, FDR corrected). The spatial pattern captured by the first eigenvariate, used to represent the within-motor-subnetwork FC, is shown in Fig. 3 and exhibits a clear division into two modules: (1) low connectivity between cortical motor regions and (2) high connectivity within subcortical regions and between subcortical and cortical motor regions. The more substantial the MDS-UPDRS decrease after DBS (capturing responders with high improvement of motor symptoms due to DBS = *optimal responders*), the more prominent this pattern of DBS OFF connectivity is. This means that the optimal responders to DBS exhibit, even in the DBS OFF state, a more prominent pattern of motor subnetwork than those with poorer response to DBS treatment. The result was found significant (ρ=-0.34, *p* = 0.040, FDR corrected) also when correcting for the effects of age, gender, PD duration, DBS duration, PD severity, and LED.

### Whole brain differences in connectivity between DBS ON and OFF states

Alterations on connection level, resulting in 4005 comparisons, in whole brain connectivity between DBS ON and OFF conditions were evaluated. Statistically significant differences were not observed, probably due to a correction to the number of connections assessed.

### The DBS ON connectivity patterns and cognition

**Fig. 4:**
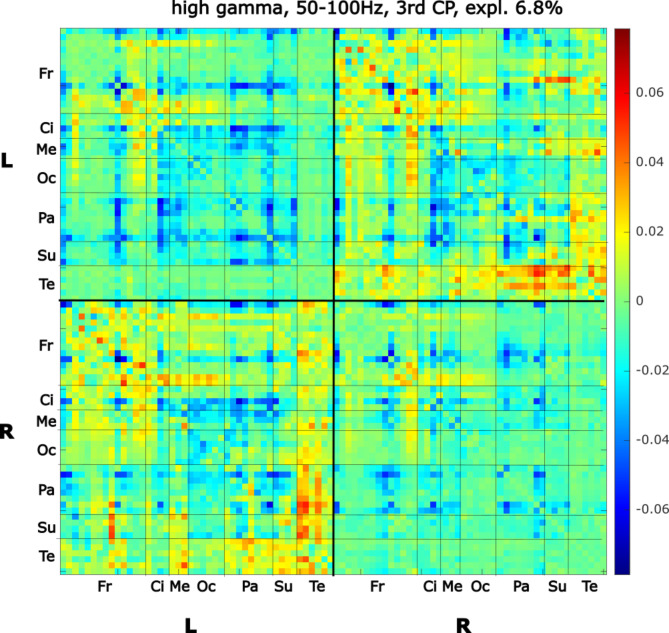
DBS ON connectivity pattern (CP_ON_) related to Stroop test interference (SI). Figure legend: CP_ON_ 3, high gamma band 50–100 Hz, explains 6.8% variability. Correlation with SI. ρ = 0.473, *p* = 0.003, uncorrected. ROIs are sorted by hemispheres; the upper left quarter shows connections within the left hemisphere (L), the lower right quarter connections within the right hemisphere (R), and the upper right and the lower left quarters show inter-hemispheric connections. Fr: Frontal, Ci: Cingulum, Me: Mesiotemporal, Oc: Occipital, Pa: Parietal, Su: Subcortical, Te: Temporal regions. Color-coded values correspond to principal component coefficients (PCA loadings): red: high PCA loadings, blue: low PCA loadings.

In examining the cognitive part of the oddball task, connectivity patterns under the DBS ON condition were compared to the cognitive scores (investigated in the DBS ON state). This provides information on which connectivity patterns reflect performance in cognitive tests. The elbow method estimated one meaningful CP in the delta band, one CP in the theta band, one in the alpha, one in the beta, two CPs in the low gamma band, and three CPs in the high gamma band. Figure 4 shows high gamma CP relevant (CP of the third principal component, ρ = 0.473, *p* = 0.003, uncorrected) to higher performance in Stroop test interference (SI) capturing the executive functions. No other cognitive score showed statistical relevance to CP_ON_s. See Supplementary Figure S4 for other CPs and Table S3 for p-values. The same result was found (ρ = 0.469, *p* = 0.004, uncorrected) when correcting for the effects of age, gender, PD duration, DBS duration, PD severity, and LED.

### The DBS OFF connectivity profile of optimal responders – classification analysis

**Fig. 5:**
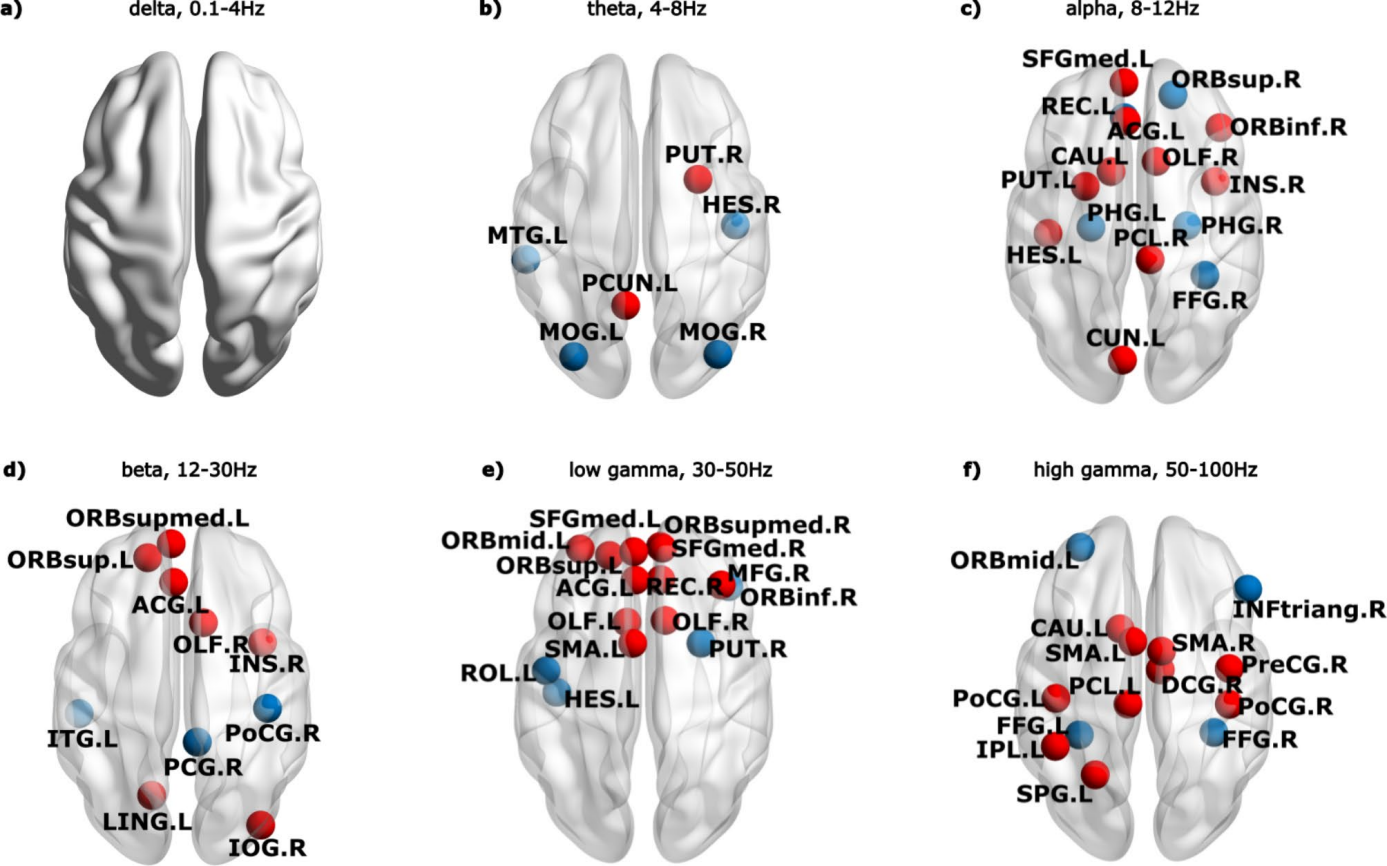
Connectivity profile discriminating optimal and suboptimal responders. Figure legend: The connectivity profile was visualized with the BrainNet Viewer^74^, http://www.nitrc.org/projects/bnv/). Red: ROIs involved in connections increasing in DBS ON compared to DBS OFF, blue: ROIs involved in connections decreasing in DBS ON compared to DBS OFF. Full ROI names are reported in Supplementary Table S4, and the matrix representation is visualized in Supplementary Figure S6.

A connectivity profile, capturing connections that are highly affected by DBS in optimal responders, was detected. Its values in the OFF state predict the level of efficacy of DBS and can be used in the future to predict a clinical response to DBS from the DBS OFF connectivity before surgery. Figure 5 shows the spatial distribution of the ROIs involved in the connectivity profile, while the connectivity matrix representation is visualized in Supplementary Figure S6. The connectivity profile spans all frequency bands except delta, with the greatest involvement of the low gamma band’s frontal connections and the high gamma band’s frontoparietal and parietal connections. A mild lateralization to the left hemisphere can be observed mainly in the gamma bands and is very probably related to the motor performance of the task by the dominant right hand in all of our subjects. The profile contains links of regions involved in the motor subnetwork – both SMAs, right Precentral Gyrus, and both Postcentral Gyri are engaged in the high gamma range, while left Caudate and left Putamen are involved in the alpha range. These connections are increasing in optimal responders in DBS ON compared to DBS OFF. The connections of the right posterior cingulate gyrus in the beta band, both parahippocampal gyri in the alpha band, and fusiform gyri in the alpha and high gamma bands also play an important role in distinguishing the level of response to DBS; in particular, they decrease in DBS ON compared to DBS OFF.

The connectivity profile classifies optimal responders with the average PPV = 0.77, NPV = 0.55, specificity = 0.73, and sensitivity = 0.60 over cross-validation folds and repetitions and was obtained at the response threshold of UPDRS_diff_<-18. The values of PPV, NPV, specificity, and sensitivity for individual folds and repetitions of the classification process are reported in Supplementary Table S5 and Supplementary Figure S7.

## Discussion

One of the suggested possibilities for improving the clinical efficacy of DBS is the refinement of the pre-implantation criteria. In the future, neurophysiological and especially electrophysiological preoperative biomarkers could help in the clinical decision-making process (for review, see^21^). Our previous work has demonstrated that network connectivity analysis may reflect the STN-DBS whole brain changes and distinguish the level of responsiveness to the therapy^26^. Many functional MRI (fMRI) studies using network connectivity have been used to predict the potential benefits of DBS therapy^50–53^. In our studies, we have analyzed surface EEG, which has significant advantages compared to MRI, such as easy availability, low costs, and better temporal resolution. The majority of previous works have used resting state data. However, this kind of data doesn’t reflect the functional performance of the examined subjects. For these reasons, we have decided to use a three-stimulus visual paradigm with both motor and cognitive load to better imitate the activities of daily living and their disease-specific limitations. The main aim of the present study was to determine DBS-related biomarkers with predictive value.

The exact mechanism of DBS functioning remains largely unknown. Mainly, the activation of the area of SMA is thought to occur via the activation of fibers within the hyper-direct pathway and to have the highest importance for the STN-DBS response in PD^54,55^. A previous crucial MRI study has described independent structural and functional connectivity predictors of clinical improvement- STN structural connectivity to the prefrontal cortex, including SMA and inferior frontal cortex and functional anticorrelation to the area of primary motor cortex^53^. The majority of frontal cortex regions provide input to STN, which then projects back to large cortical regions through the thalamus^56^, but which of these many connections could be associated with maximal DBS effects on motor symptoms remains unknown.

Compared to MRI, one of the advantages of EEG analysis is the ability to study well-known PD-related oscillopathies with clear pathophysiological significance. Hypersynchronized beta oscillations in motor circuits have been shown to correlate to bradykinesia and rigidity^13,14,16^. Gamma power is reduced in PD; this reduction correlates with the main motor symptoms, similar to beta hypersynchrony, and is restored after medication and DBS^19^. Alpha and theta activity power changes have been shown to correlate mainly with non-motor symptoms and functions^24,26,57–60^.

In the current study, we first analyzed connectivity changes in the motor circuits, as DBS should primarily influence these areas. Looking into the subcortical and cortical regions involved in motor control, a significant increase in the representative connectivity of this subnetwork was measured in the high gamma band, 50–100 Hz, in DBS ON compared to DBS OFF. Specifically, as revealed by the post hoc testing, this increase reflects significant increases in the connectivity of both the Precentral gyri and four regions in the left hemisphere: SMA, Postcentral gyrus, Caudate, and Putamen. As the gamma band power is known to have a prokinetic effect^61,62^ and the motor task was performed by the dominant right hand, this is not an unexpected result – see Supplementary Figure S2. It can be seen in Figure S2 that the interhemispheric connectivity in motor circuits is stronger than the intrahemispheric within different motor structures. This is probably related to the pathophysiology of the disease. Disturbed mainly beta and gamma reactivity and effective lateralization have been described during movement performance in PD^63,64^. DBS increases both interhemispheric and intrahemispheric connectivity- see Figure S2. On the interhemispheric level, it is mainly the area of the postcentral gyrus (primary somatosensory cortex) that is known to be involved in the preparation and execution phases of movement, including gait (for review, see^65^). On the intrahemispheric level, DBS increases connectivity between SMA, the primary motor cortex, and the basal ganglia in the left hemisphere. We believe that these changes are related to the clinical improvement of the main motor PD symptoms.

Analyzing the correlation of DBS OFF within-motor subnetwork connectivity with motor improvement, measured by MDS-UPDRS_diff_, provides a way to associate the motor subnetwork with the quantitative effect of DBS treatment. The MDS-UPDRS_diff_ was also used in this paper as a marker to differentiate optimal responders from others. A high gamma DBS OFF within-motor subnetwork connectivity correlated with the movement improvement caused by DBS. The higher the improvement of the motor symptoms, the more prominent the relevant spatial pattern (captured by the first principal component used to represent within-motor subnetwork connectivity) of high connectivity within basal ganglia and thalamus and between subcortical and cortical motor areas and low connectivity within cortical motor regions – see Fig. 3.

On the level of individual connections of the whole brain, statistically significant changes across all frequency bands were not observed in the DBS ON condition compared to DBS OFF. The effect of DBS on cognition has been extensively studied; for the review, see, for example^66,67^, . Overall, mixed findings were reported for both acute and long-term high-frequency DBS. The most frequent declines were reported in executive functioning, including verbal fluency, working memory, and planning. However, the visuospatial ability was contrarily reported to improve with DBS, while no effect on memory was observed^68^. Concerning the cognitive part of the motor-cognitive task in the current study, a correlation between the Stroop interference test and one connectivity pattern was detected. A high score on the SI test was found to be related to the prominent pattern of high connectivity of predominantly right frontal areas with the left hemisphere in the high gamma band, low intra-hemispheric connectivity of mesiotemporal, occipital, parietal, and subcortical regions, and the increased connectivity of the left temporal regions with the areas in the right hemisphere. As the correlation of DBS ON connectivity pattern with cognitive performance suggests, the clinical factors are linked more with the prominent positive patterns of interhemispheric connections and negative within-hemispheric functional connectivity.

The first papers on MR connectivity^53,69^ identifying connectivity profiles of positive DBS outcomes in PD link these to electrode placements. The current study proposes an electrophysiological profile to predict the clinical effect of DBS in PD. It spans all frequency bands except the delta band, with important connections in sensorimotor/frontal, parietal, and subcortical regions. Our findings can be loosely compared to a recent resting-state fMRI paper reporting increased functional connectivity between subcortical and frontal structures being strong indicators of positive DBS outcome^70^. The classifier’s quality expressed by PPV and a specificity of around 75% suggests a good ability to capture responders that substantially benefit from DBS. However, poor results in the NPV and sensitivity around 60% imply quite a large number of optimal responders classified as suboptimal. This leads to a belief that the proposed connectivity profile either fails to cover the whole spectrum of connectivity features to a great enough degree to be promising or that some of its connections do not play a role in mediating the treatment. However, when combined with the whole-brain connectivity pattern related to the cognitive tests and the motor subnetwork pattern related to the improvement of clinical symptoms (MDS-UDPRS), it can inform about functional circuits relevant to an optimal response to DBS.

## Limitations

We are aware that the short-lasting DBS-OFF condition after several months or years of DBS treatment does not correspond to the preoperative parkinsonian pathophysiological state. Future studies on preoperative EEG data are necessary to verify the potential clinical usefulness of the connectivity profile analysis to predict the future DBS treatment response.

Our approach did not have the power to detect the normalization of beta-band hypersynchrony, a previously proven effect of deep brain stimulation. It is indisputable, mainly in the area of basal ganglia, but probably not significant on the whole brain level recorded from the scalp, as the gamma band power is also known to be the primary correlate of activation with a prokinetic significance in cortical areas. Also, the quality of the proposed classification connectivity profile has to be improved to be applicable in the clinical practice.

## Conclusions and future work

We have detected specific (visual-motor-cognitive) HDEEG connectivity patterns in the high gamma band (50–100 Hz); one is related to the clinical main PD motor symptoms improvement after DBS in the motor subcortico-cortical subnetwork areas, the other is related to cognition on the whole brain level. Finally, a whole brain multifrequency connectivity profile was found to be a potential marker with a predictive value for therapy responsiveness. This analysis provided an explicit hypothesis on the localization of the key functional connections and patterns thereof, which focused replication studies can target in the future.

We plan to implement the results of this study to preoperative HD-EEG to confirm or disprove their significance. Our results and significant connectivity patterns could also be compared to DBS simulation studies of the beta power changes^56,71–73^ or provide additions for modeling motor-cognitive aspects of PD and the influence of DBS.

## Electronic supplementary material

Below is the link to the electronic supplementary material.

Supplementary Material 1

## Data Availability

The data that support the findings of this study are available from the corresponding author upon request.

## References

[1] for Parkinson’s Disease Study Group. Deep-brain stimulation of the subthalamic nucleus or the pars interna of the Globus Pallidus in Parkinson’s disease. *N. Engl. J. Med.* **345**, 956–963 (2001). 11575287 10.1056/NEJMoa000827

[2] Rodriguez-Oroz, M. C. et al. Bilateral deep brain stimulation in Parkinson’s disease: A multicentre study with 4 years follow-up. *Brain* **128**, 2240–2249 (2005). 15975946 10.1093/brain/awh571

[3] Deuschl, G. et al. A randomized trial of deep-brain stimulation for Parkinson’s disease. *N. Engl. J. Med.* **355**, 896–908 (2006). 16943402 10.1056/NEJMoa060281

[4] Moro, E. et al. Long-term results of a multicenter study on subthalamic and pallidal stimulation in Parkinson’s disease. *Mov. Disord.* **25**, 578–586 (2010). 20213817 10.1002/mds.22735

[5] Lozano, A. M. et al. Deep brain stimulation: Current challenges and future directions. *Nat. Rev. Neurol.* **15**, 148–160 (2019). 30683913 10.1038/s41582-018-0128-2 PMC6397644

[6] Temel, Y. et al. Behavioural changes after bilateral subthalamic stimulation in advanced Parkinson disease: A systematic review. *Parkinsonism Relat. Disord* **12**, 265–272 (2006). 16621661 10.1016/j.parkreldis.2006.01.004

[7] Voon, V., Kubu, C., Krack, P., Houeto, J. L. & Tröster A. I. Deep brain stimulation: Neuropsychological and neuropsychiatric issues. *Mov. Disord* **21**, S305–S327 (2006). 16810676 10.1002/mds.20963

[8] Witt, K. et al. Neuropsychological and psychiatric changes after deep brain stimulation for Parkinson’s disease: A randomised, multicentre study. *Lancet Neurol.* **7**, 605–614 (2008). 18538636 10.1016/S1474-4422(08)70114-5

[9] Little, S. et al. Adaptive deep brain stimulation in advanced Parkinson disease. *Ann. Neurol.* **74**, 449–457 (2013). 23852650 10.1002/ana.23951 PMC3886292

[10] Habets, J. G. V. et al. An update on adaptive deep brain stimulation in Parkinson’s disease. *Mov. Disord.* **33**, 1834–1843 (2018). 30357911 10.1002/mds.115 PMC6587997

[11] Tinkhauser, G. et al. The modulatory effect of adaptive deep brain stimulation on beta bursts in Parkinson’s disease. *Brain* **140**, 1053–1067 (2017). 28334851 10.1093/brain/awx010 PMC5382944

[12] Alonso-Frech, F. et al. Slow oscillatory activity and levodopa-induced dyskinesias in Parkinson’s disease. *Brain* **129**, 1748–1757 (2006). 16684788 10.1093/brain/awl103

[13] Chen, C. C. et al. Complexity of subthalamic 13–35 hz oscillatory activity directly correlates with clinical impairment in patients with Parkinson’s disease. *Exp. Neurol.* **224**, 234–240 (2010). 20353774 10.1016/j.expneurol.2010.03.015

[14] Oswal, A. et al. Deep brain stimulation modulates synchrony within spatially and spectrally distinct resting state networks in Parkinson’s disease. *Brain* **139**, 1482–1496 (2016). 27017189 10.1093/brain/aww048 PMC4845255

[15] Steiner, L. A. et al. Subthalamic beta dynamics mirror parkinsonian bradykinesia months after neurostimulator implantation. *Mov. Disord.* **32**, 1183–1190 (2017). 28639263 10.1002/mds.27068 PMC5575541

[16] Kühn, A. A., Kupsch, A., Schneider, G. H. & Brown, P. Reduction in subthalamic 8–35 hz oscillatory activity correlates with clinical improvement in Parkinson’s disease. *Eur. J. Neurosci.* **23**, 1956–1960 (2006). 16623853 10.1111/j.1460-9568.2006.04717.x

[17] van Wijk, B. C. M. et al. Subthalamic nucleus phase–amplitude coupling correlates with motor impairment in Parkinson’s disease. *Clin. Neurophysiol.* **127**, 2010–2019 (2016). 26971483 10.1016/j.clinph.2016.01.015 PMC4803022

[18] Bočková, M. et al. Coupling between beta band and high frequency oscillations as a clinically useful biomarker for DBS. *NPJ Parkinsons Dis.* **10**, 40 (2024). 38383550 10.1038/s41531-024-00656-8 PMC10882016

[19] López-Azcárate, J. et al. Coupling between beta and high-frequency activity in the human subthalamic nucleus may be a pathophysiological mechanism in Parkinson’s disease. *J. Neurosci.* **30**, 6667–6677 (2010). 20463229 10.1523/JNEUROSCI.5459-09.2010 PMC6632566

[20] Litvak, V., Florin, E., Tamás, G., Groppa, S. & Muthuraman, M. EEG and MEG primers for tracking DBS network effects. *Neuroimage* **224**, 117447 (2021). 33059051 10.1016/j.neuroimage.2020.117447

[21] Bočková, M. & Rektor, I. Impairment of brain functions in Parkinson’s disease reflected by alterations in neural connectivity in EEG studies: A viewpoint. *Clin. Neurophysiol.* **130**, 239–247 (2019). 30580247 10.1016/j.clinph.2018.11.013

[22] Markser, A. et al. Deep brain stimulation and cognitive decline in Parkinson’s disease: The predictive value of electroencephalography. *J. Neurol.* **262**, 2275–2284 (2015). 26159102 10.1007/s00415-015-7839-8

[23] Yakufujiang, M. et al. Predictive potential of preoperative electroencephalogram for neuropsychological change following subthalamic nucleus deep brain stimulation in Parkinson’s disease. *Acta Neurochir. (Wien)* **161**, 2049–2058 (2019). 31278598 10.1007/s00701-019-03991-5

[24] Onofrj, M., Espay, A. J., Bonanni, L., Delli Pizzi, S. & Sensi, S. L. Hallucinations, somatic-functional disorders of PD-DLB as expressions of thalamic dysfunction. *Mov. Disord.* **34**, 1100–1111 (2019). 31307115 10.1002/mds.27781 PMC6707070

[25] Geraedts, V. J. et al. Machine learning for automated EEG-based biomarkers of cognitive impairment during deep brain stimulation screening in patients with Parkinson’s Disease. *Clin. Neurophysiol.* **132**, 1041–1048 (2021). 33743299 10.1016/j.clinph.2021.01.021

[26] Bočková, M. et al. Cortical network organization reflects clinical response to subthalamic nucleus deep brain stimulation in Parkinson’s disease. *Hum. Brain Mapp.* **42**, 5626–5635 (2021). 34448523 10.1002/hbm.25642 PMC8559467

[27] Polich, J. Updating P300: An integrative theory of P3a and P3b. *Clin. Neurophysiol.* **118**, 2128–2148 (2007). 17573239 10.1016/j.clinph.2007.04.019 PMC2715154

[28] Bočková, M. et al. Oscillatory changes in cognitive networks activated during a three-stimulus visual paradigm: An intracerebral study. *Clin. Neurophysiol.* **124**, 283–291 (2013). 22938795 10.1016/j.clinph.2012.07.009

[29] Fournier, L. R. et al. Which task will we choose first? Precrastination and cognitive load in task ordering. *Atten. Percept. Psychophys* **81**, 489–503 (2019). 30506327 10.3758/s13414-018-1633-5

[30] Horn, A. & Kühn, A. A. Lead-DBS: A toolbox for deep brain stimulation electrode localizations and visualizations. *Neuroimage* **107**, 127–135 (2015). 25498389 10.1016/j.neuroimage.2014.12.002

[31] Ewert, S. et al. Toward defining deep brain stimulation targets in MNI space: A subcortical atlas based on multimodal MRI, histology and structural connectivity. *Neuroimage* **170**, 271–282 (2018). 28536045 10.1016/j.neuroimage.2017.05.015

[32] Delorme, A. & Makeig, S. EEGLAB: An open source toolbox for analysis of single-trial EEG dynamics including independent component analysis. *J. Neurosci. Methods* **134**, 9–21 (2004). 15102499 10.1016/j.jneumeth.2003.10.009

[33] Coito, A., Michel, C. M., Vulliemoz, S. & Plomp, G. Directed functional connections underlying spontaneous brain activity. *Hum. Brain Mapp.* **40**, 879–888 (2019). 30367722 10.1002/hbm.24418 PMC6865461

[34] Zaldivar, D., Goense, J., Lowe, S. C., Logothetis, N. K. & Panzeri, S. Dopamine is signaled by mid-frequency oscillations and boosts output layers visual information in visual cortex. *Curr. Biol.* **28**, 224–235 (2018). 29307559 10.1016/j.cub.2017.12.006

[35] Plesinger, F., Jurco, J., Halamek, J. & Jurak, P. SignalPlant: An open signal processing software platform. *Physiol. Meas.* **37**, N38 (2016). 27243208 10.1088/0967-3334/37/7/N38

[36] Lio, G., Thobois, S., Ballanger, B., Lau, B. & Boulinguez, P. Removing deep brain stimulation artifacts from the electroencephalogram: Issues, recommendations and an open-source toolbox. *Clin. Neurophysiol.* **129**, 2170–2185 (2018). 30144660 10.1016/j.clinph.2018.07.023

[37] Lamoš, M. et al. The effect of deep brain stimulation in Parkinson’s disease reflected in EEG microstates. *NPJ Parkinsons Dis.* **9**, 63 (2023). 37069159 10.1038/s41531-023-00508-x PMC10110608

[38] Bočková, M. et al. Suboptimal response to STN-DBS in Parkinson’s disease can be identified via reaction times in a motor cognitive paradigm. *J. Neural Transm* **127**, 1579–1588 (2020). 32965592 10.1007/s00702-020-02254-3

[39] Chaumon, M., Bishop, D. V. M. & Busch, N. A. A practical guide to the selection of independent components of the electroencephalogram for artifact correction. *J. Neurosci. Methods* **250**, 47–63 (2015). 25791012 10.1016/j.jneumeth.2015.02.025

[40] Brunet, D., Murray, M. M. & Michel, C. M. Spatiotemporal analysis of multichannel EEG: CARTOOL. *Comput Intell Neurosci* 1–15 (2011). (2011). 10.1155/2011/813870 PMC3022183 21253358

[41] Tzourio-Mazoyer, N. et al. Automated anatomical labeling of activations in SPM using a macroscopic anatomical parcellation of the MNI MRI single-subject brain. *Neuroimage* **15**, 273–289 (2002). 11771995 10.1006/nimg.2001.0978

[42] Coito, A., Michel, C. M., Van Mierlo, P., Vulliemoz, S. & Plomp, G. Directed functional brain connectivity based on EEG source imaging: Methodology and application to temporal lobe epilepsy. *IEEE Trans. Biomed. Eng.* **63**, 2619–2628 (2016). 27775899 10.1109/TBME.2016.2619665

[43] Litvak, V. et al. Movement-related changes in local and long-range synchronization in Parkinson’s disease revealed by simultaneous magnetoencephalography and intracranial recordings. *J. Neurosci.* **32**, 10541–10553 (2012). 22855804 10.1523/JNEUROSCI.0767-12.2012 PMC3428626

[44] Wiest, C. et al. Finely-tuned gamma oscillations: Spectral characteristics and links to dyskinesia. *Exp. Neurol.* **351**, 113999 (2022). 35143832 10.1016/j.expneurol.2022.113999 PMC7612436

[45] Seeber, M. et al. Subcortical electrophysiological activity is detectable with high-density EEG source imaging. *Nat. Commun.* **10**, 753 (2019). 30765707 10.1038/s41467-019-08725-w PMC6376013

[46] Stam, C. J., Nolte, G. & Daffertshofer, A. Phase lag index: Assessment of functional connectivity from multi channel EEG and MEG with diminished bias from common sources. *Hum. Brain Mapp.* **28**, 1178–1193 (2007). 17266107 10.1002/hbm.20346 PMC6871367

[47] Cohen, M. X. Effects of time lag and frequency matching on phase-based connectivity. *J. Neurosci. Methods* **250**, 137–146 (2015). 25234308 10.1016/j.jneumeth.2014.09.005

[48] Birot, G. et al. Head model and electrical source imaging: A study of 38 epileptic patients. *Neuroimage Clin.* **5**, 77–83 (2014). 25003030 10.1016/j.nicl.2014.06.005 PMC4081973

[49] Michel, C. M. & Brunet, D. EEG source imaging: A practical review of the analysis steps. *Front. Neurol.* **10**, 325 (2019). 31019487 10.3389/fneur.2019.00325 PMC6458265

[50] Kahan, J. et al. Resting state functional MRI in Parkinson’s disease: The impact of deep brain stimulation on ‘effective’connectivity. *Brain* **137**, 1130–1144 (2014). 24566670 10.1093/brain/awu027 PMC3959559

[51] Middlebrooks, E. H. et al. Differences in functional connectivity profiles as a predictor of response to anterior thalamic nucleus deep brain stimulation for epilepsy: A hypothesis for the mechanism of action and a potential biomarker for outcomes. *Neurosurg. Focus* **45**, E7 (2018). 30064322 10.3171/2018.5.FOCUS18151

[52] Younce, J. R. et al. Resting-state functional connectivity predicts STN DBS Clinical Response. *Mov. Disord.* **36**, 662–671 (2021). 33211330 10.1002/mds.28376 PMC7987812

[53] Horn, A. et al. Connectivity predicts deep brain stimulation outcome in P arkinson disease. *Ann. Neurol.* **82**, 67–78 (2017). 28586141 10.1002/ana.24974 PMC5880678

[54] Schneider, L., Seeger, V., Timmermann, L. & Florin, E. Electrophysiological resting state networks of predominantly akinetic-rigid Parkinson patients: Effects of dopamine therapy. *Neuroimage Clin.* **25**, 102147 (2020). 31954989 10.1016/j.nicl.2019.102147 PMC6965744

[55] Horn, A., Neumann, W. J., Degen, K., Schneider, G. H. & Kühn, A. A. Toward an electrophysiological sweet spot for deep brain stimulation in the subthalamic nucleus. *Hum. Brain Mapp.* **38**, 3377–3390 (2017). 28390148 10.1002/hbm.23594 PMC6867148

[56] Sobesky, L. et al. Subthalamic and pallidal deep brain stimulation: Are we modulating the same network? *Brain* **145**, 251–262 (2022). 34453827 10.1093/brain/awab258

[57] Fumagalli, M. et al. Conflict-dependent dynamic of subthalamic nucleus oscillations during moral decisions. *Soc. Neurosci.* **6**, 243–256 (2011). 21061226 10.1080/17470919.2010.515148

[58] Huebl, J. et al. Oscillatory subthalamic nucleus activity is modulated by dopamine during emotional processing in Parkinson’s disease. *Cortex* **60**, 69–81 (2014). 24713195 10.1016/j.cortex.2014.02.019

[59] Welter, M. L. et al. Basal ganglia dysfunction in OCD: Subthalamic neuronal activity correlates with symptoms severity and predicts high-frequency stimulation efficacy. *Transl Psychiatry* **1**, e5–e5 (2011). 22832400 10.1038/tp.2011.5 PMC3309476

[60] Rappel, P. et al. Subthalamic theta activity: A novel human subcortical biomarker for obsessive compulsive disorder. *Transl Psychiatry* **8**, 118 (2018). 29915200 10.1038/s41398-018-0165-z PMC6006433

[61] Brown, P. Oscillatory nature of human basal ganglia activity: Relationship to the pathophysiology of Parkinson’s disease. *Mov. Disord* **18**, 357–363 (2003). 12671940 10.1002/mds.10358

[62] Androulidakis, A. G. et al. Dopaminergic therapy promotes lateralized motor activity in the subthalamic area in Parkinson’s disease. *Brain* **130**, 457–468 (2007). 17213215 10.1093/brain/awl358

[63] Doyle, L. M. F. et al. Levodopa-induced modulation of subthalamic beta oscillations during self-paced movements in patients with Parkinson’s disease. *Eur. J. Neurosci.* **21**, 1403–1412 (2005). 15813950 10.1111/j.1460-9568.2005.03969.x

[64] Kühn, A. A. et al. Event-related beta desynchronization in human subthalamic nucleus correlates with motor performance. *Brain* **127**, 735–746 (2004). 14960502 10.1093/brain/awh106

[65] Abbruzzese, G. & Berardelli, A. Sensorimotor integration in movement disorders. *Mov. Disord.* **18**, 231–240 (2003). 12621626 10.1002/mds.10327

[66] Cole, R. C., Okine, D. N., Yeager, B. E. & Narayanan, N. S. Neuromodulation of cognition in Parkinson’s disease. *Prog Brain Res.* **269**, 435–455 (2022). 35248205 10.1016/bs.pbr.2022.01.016 PMC9199111

[67] Brittain, J. S. & Cagnan, H. Recent trends in the Use of Electrical Neuromodulation in Parkinson’s Disease. *Curr. Behav. Neurosci. Rep.* **5**, 170–178 (2018). 29862163 10.1007/s40473-018-0154-9 PMC5962624

[68] You, Z. et al. Efforts of subthalamic nucleus deep brain stimulation on cognitive spectrum: From explicit to implicit changes in the patients with Parkinson’s disease for 1 year. *CNS Neurosci. Ther.* **26**, 972–980 (2020). 32436660 10.1111/cns.13392 PMC7415202

[69] Vanegas-Arroyave, N. et al. Tractography patterns of subthalamic nucleus deep brain stimulation. *Brain* **139**, 1200–1210 (2016). 26921616 10.1093/brain/aww020 PMC5006230

[70] Albano, L. et al. Functional connectivity in Parkinson’s disease candidates for deep brain stimulation. *NPJ Parkinsons Dis.* **8**, 4 (2022). 35013326 10.1038/s41531-021-00268-6 PMC8748462

[71] Yousif, N., Bain, P. G., Nandi, D. & Borisyuk, R. A population model of deep brain stimulation in movement disorders from circuits to cells. *Front. Hum. Neurosci.* **14**, 55 (2020). 32210779 10.3389/fnhum.2020.00055 PMC7066497

[72] Meier, J. M. et al. Virtual deep brain stimulation: Multiscale co-simulation of a spiking basal ganglia model and a whole-brain mean-field model with the virtual brain. *Exp. Neurol.* **354**, 114111 (2022). 35569510 10.1016/j.expneurol.2022.114111

[73] Maith, O. et al. A computational model-based analysis of basal ganglia pathway changes in Parkinson’s disease inferred from resting-state fMRI. *Eur. J. Neurosci.* **53**, 2278–2295 (2021). 32558966 10.1111/ejn.14868

[74] Xia, M., Wang, J. & He, Y. BrainNet Viewer: A network visualization tool for human brain connectomics. *PLoS One* **8**, e68910 (2013). 23861951 10.1371/journal.pone.0068910 PMC3701683

