# Supplementary material for: Revealing connectivity patterns of deep brain stimulation efficacy in Parkinson’s disease
Source: Sci Rep. 2024 Dec 30;14:31652. doi: 10.1038/s41598-024-80630-9 (PMC11686061; doi:10.1038/s41598-024-80630-9)
Supplement: Supplementary file 1 — Supplementary Material 1 [file 41598_2024_80630_MOESM1_ESM.pdf]

Supplementary material

Revealing Connectivity Patterns of Deep Brain Stimulation Efficacy in Parkinson's Disease

Eva Výtvarová, Martin Lamoš, Jaroslav Hlinka, Sabina Goldemundová, Ivan Rektor, Martina Bočková

Table S1: Patient characteristics.

No.	age	sex	PD/DBS duration (years)	DBS settings	Medication, LED (mg)	MDS-UPDRS OFF	MDS-UPDRS ON
1	65	F	11/4	2.5mA bilat/130Hz/91μs (Libra)	L-dopa, Ent, Rop, 1012	28	11
2	71	M	8/2	3.0V bilat/130Hz/ 90μs (Activa)	L-dopa, Rop, 1080	45	25
3	68	F	10/1.5	3.2V bilat/130Hz/90μs (Activa)	L-dopa, Ent, Rop, 1052	34	17
4	58	M	25/3	1.1mA bilat/130Hz/143μs (Libra)	L-dopa, Rop, 940	40	26
5	52	M	12/2	3.3V bilat/130Hz/90μs (Activa)	L-dopa, Rot, 1480	42	14
6	55	M	6/1	1.5V bilat/130Hz/60μs (Activa)	L-dopa, Pra, 1044	46	29
7	64	F	11/2	2.4V bilat/130Hz/90μs (Activa)	L-dopa, Ent, 493	50	17
8	61	F	12/1	2.4V bilat/130Hz/90μs (Activa)	L-dopa, Ent, Rop, 1079	35	15
9	39	M	8/1	3.9mA/3.0mA(bip)/130Hz/78μs (Libra)	L-dopa, Ent, Rop, 626	48	13
10	60	M	7/0.5	1.9V bilat/130Hz/90μs (Activa)	L-dopa, Ent, 1596	31	18
11	65	F	12/2	3.2mA bilat (bip)/130Hz/65μs (Libra)	L-dopa, Rop, 910	44	31
12	53	M	9/1	2.2 V bilat/130Hz/90μs (Activa)	L-dopa, Ent, Rop, 919	48	28
13	58	M	19/8	3.3V/4.3V/130Hz/90μs (Activa)	L-dopa, Rop, Rot, Bipe, 1300	53	37
14	55	F	8/1	1.4mA/1.8mA/130Hz/91μs (Libra)	L-dopa, Ent, Pra, 487	55	31
15	65	F	10/1	1.6V/1.4V/130Hz/90μs (Activa)	L-dopa, Pra, 406	36	17
16	56	M	8/2	2.1V/2.1V(bip)/130Hz/90μs (Activa)	L-dopa, Ent, 1119	30	17
17	58	M	14/1	1.2V/2.5V/130Hz/90μs (Activa)	L-dopa, Ent, Rop, 659	49	26
18	63	M	9/3	2.1V bilat/90μs/130Hz (Activa)	L-dopa, Ent, Rop, Sel, 1351	24	15
19	66	M	10/2	2.5V bilat/90μs/130Hz (Activa)	L-dopa, Ent, Rop, 839	50	24
20	73	M	18/7	2.5V/ 3.0V /90μs/130Hz (Activa)	L-dopa, Ent, 1357	30	11
21	59	M	8/2	2.6V bilat/90μs/130Hz (Activa)	L-dopa, 750	40	21
22	56	M	10/3	3.0V bilat/60μs/130Hz (Activa)	L-dopa, Ent, 1330	36	21
23	64	M	10/1	2.2V bilat/90μs/130Hz (Activa)	L-dopa, Rop, 879	38	14
24	66	M	9/2	2.8V bilat /90μs/130Hz (Activa)	L-dopa, Pra, 1210	36	25
25	54	M	10/3	3.6V bilat /60μs/130Hz (Activa)	L-dopa, Ent, Rop, 1224	36	17
26	65	M	18/2	1.4V bilat /90μs/130Hz (Activa)	L-dopa, Pra, 456	44	24
27	68	F	18/2	2.2mA bilat /91μs/130Hz (Libra)	L-dopa, Ent, Ama, 1098	29	9
28	66	M	8/1	2.9V and 2.7V/90μs/130Hz (Activa)	L-dopa, Ent, 1164	36	21
29	54	M	16/8	3.2V bilat /90μs/130Hz (Activa)	L-dopa, Ent, Rop, Ama, 1413	58	24
30	68	F	16/0.5	0.5 and 1.7V/90μs/130Hz (Activa)	L-dopa, Rop, 1445	42	23
31	64	M	12/1	2.6V bilat /90μs/130Hz (Activa)	L-dopa, Ent, Rop, 1052	64	33
32	58	M	17/0.5	1.0V bilat /90μs/180Hz (Activa)	L-dopa, Ent, Pra, 609	26	17
33	69	M	7/1	3.3V bilat /90μs/130Hz (Activa)	0 (severe glaucoma)	39	30
34	66	M	17/1.5	1.5V/4.0V bip/90μs/140Hz (Activa)	L-dopa, Tol, Rot, Ama, 340	41	27
35	68	F	10/1	1.4mA bilat/60μs/130Hz (Percept)	L-dopa, Ent, Rop, 670	48	37
36	65	F	22/3	1.7 V bilat/90μs/130Hz (Activa PC)	L-dopa, Ent, Pra, 609	35	12
37	51	M	8/1	2.5mA and 3.5mA /90μs/110Hz (Percept)	L-dopa, 400	50	9
38	66	M	11/3	1.6V bilat/90μs/130 Hz (Activa PC)	L-dopa, Rot, 980	30	8
39	58	F	8/1	2.3mA bilat/90μs/130 Hz (Infinity)	L-dopa, 450	38	15
40	49	M	10/1.5	3.7 mA and 2.8mA/60μs/130Hz (Percept)	L-dopa, Ent, 1281	60	28
41	64	M	10/1	1.5 mA bilat /90μs/130 Hz (Infinity)	L-dopa, Ent, Pra, 1103	28	15
42	65	F	11/1	1.6mA and 1.3 mA/90μs/130Hz (Percept)	L-dopa, 775	53	24
43	58	F	3.5/1.5	1.1mA bilat/90μs/130 Hz (Percept)	0 (tremor dominant, medication resistant)	0	0

Bilat- bilaterally, L-dopa- levodopa, Ent- entacapone, Rop- ropinirole, Pra- pramipexole, Tol- tolcapone, Rot- rotigotine, Ama- amantadine, Bipe- biperiden, Sel- selegiline

Table S2: Characteristics of the patient group. Average values ± standard deviations. [*]Three subjects were forced right-handed.

age	61.07 ± 6.76	Digit span	45.14 ± 9.19
sex	14 females, 29 males	Stroop interference	47.60 ± 10.60
Right-handed/left-handed	43 / 0[*]	Word list 1	46.07 ± 12.33
MDS-UPDRS$_{OFF}$	40.12 ± 11.50	Word list 2	53.62 ± 7.56
MDS-UPDRS$_{ON}$	20.37 ± 8.21	Mattis total score	140.64 ± 3.61

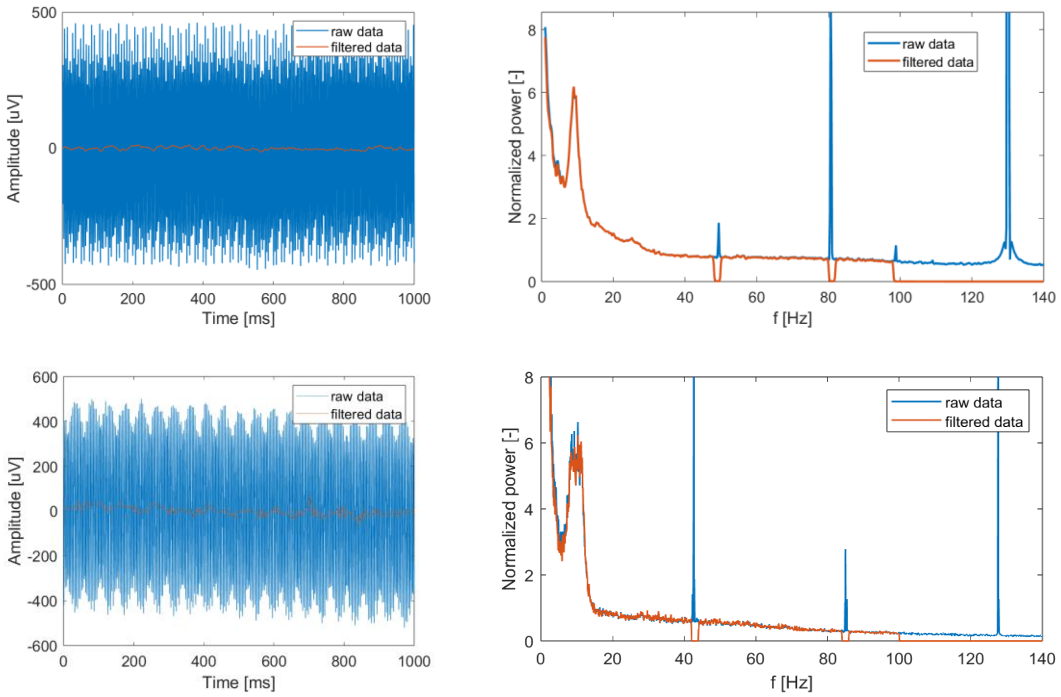

Figure S1: Example of DBS artifact suppression in two subjects under the DBS ON condition: time domain (left) and power spectrum (right) of raw (in blue) and filtered (in orange) signal from one electrode.

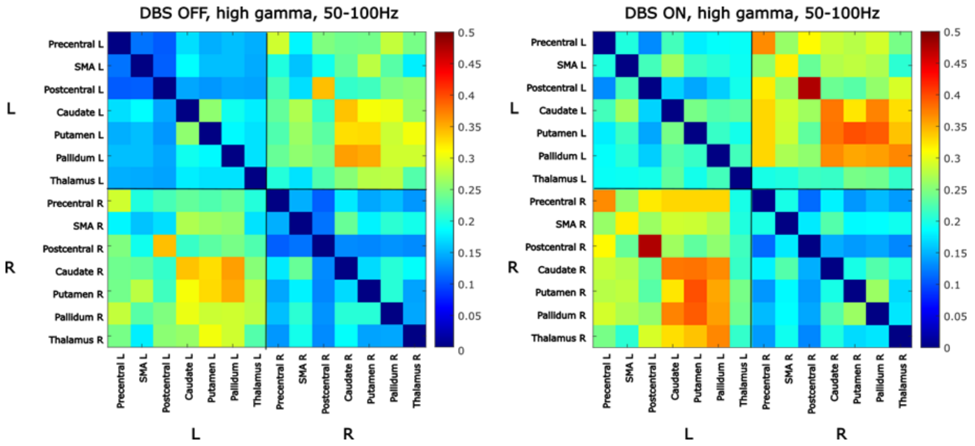

Figure S2: The average (across subjects) connectivity within the motor subnetwork in DBS OFF and DBS ON states, high gamma band, 50-100Hz. ROIs are sorted by hemispheres; the upper left quarter shows connections within the left hemisphere, the lower right quarter connections within the right hemisphere, and the upper right and the lower left quarters show inter-hemispheric connections.

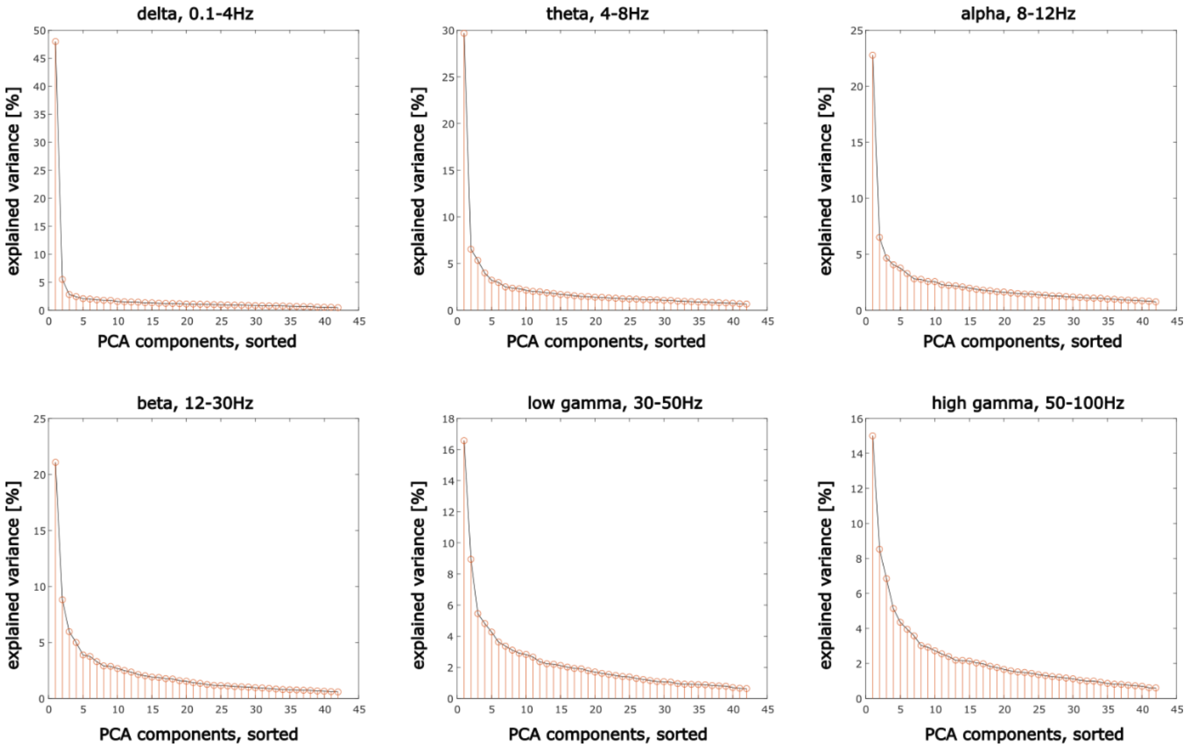

Figure S3: Functions of explained variance by individual PCA components of DBS ON whole-brain connectivity. By an elbow criterion, these numbers of components were used to define DBS ON connectivity patterns CP_{ON}s: 1 for delta, 1 for theta, 1 for alpha, 1 for beta, 2 for low gamma, and 3 for high gamma band.

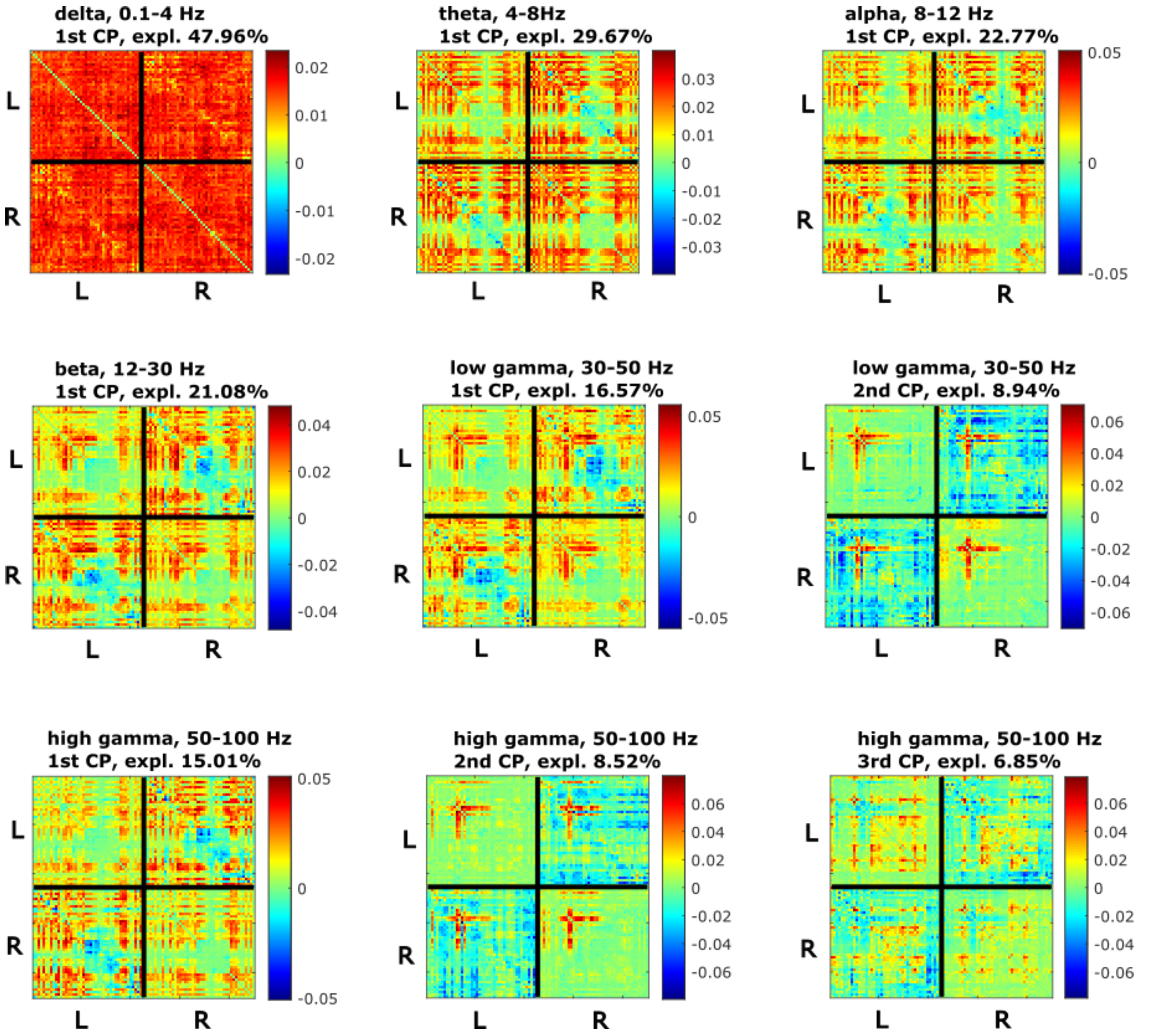

Figure S4: DBS ON connectivity patterns (CP_{ON}s). ROIs are sorted by hemispheres; the upper left quarter shows connections within the left hemisphere, the lower right quarter connections within the right hemisphere, and the upper right and the lower left quarters show inter-hemispheric connections. Color-coded values correspond to PCA loadings: red: higher connectivity, blue: lower connectivity.

Table S3: P-values of correlations between DBS ON connectivity profiles CPs and cognitive scores. Bold: Significant results that are reported in the Results section of the full paper. DS: Digit span, WL1: Word list 1, WL2: Word list 2, SI: Stroop interference, MT: Mattis total score.

**DS**	**1st CP_{ON}**	**2nd CP_{ON}**	**3rd CP_{ON}**
**delta**	p=0.27	-	-
**theta**	p=0.65	-	-
**alpha**	p=0.91	-	-
**beta**	p=0.40	-	-
**low gamma**	p=0.48	p=0.78	-
**high gamma**	p=0.91	p=0.65	p=0.08
**WL1**	**1st CP_{ON}**	**2nd CP_{ON}**	**3rd CP_{ON}**
**delta**	p=0.50	-	-
**theta**	p=0.22	-	-
**alpha**	p=0.13	-	-
**beta**	p=0.42	-	-
**low gamma**	p=0.78	p=0.79	-
**high gamma**	p=0.79	p=0.46	p=0.97
**WL2**	**1st CP_{ON}**	**2nd CP_{ON}**	**3rd CP_{ON}**
**delta**	p=0.92	-	-
**theta**	p=0.61	-	-
**alpha**	p=0.10	-	-
**beta**	p=0.85	-	-
**low gamma**	p=0.76	p=0.88	-
**high gamma**	p=0.90	p=0.29	p=0.21
**SI**	**1st CP_{ON}**	**2nd CP_{ON}**	**3rd CP_{ON}**
**delta**	p=0.24	-	-
**theta**	p=0.65	-	-
**alpha**	p=0.73	-	-
**beta**	p=0.47	-	**-**
**low gamma**	p=0.69	p=0.75	-
**high gamma**	p=0.05	p=0.46	**p=0.003**
**MT**	**1st CP_{ON}**	**2nd CP_{ON}**	**3rd CP_{ON}**
**delta**	p=0.52	-	-
**theta**	p=0.50	-	-
**alpha**	p=0.13	-	-
**beta**	p=0.76	-	-
**low gamma**	p=0.59	p=0.77	-
**high gamma**	p=0.50	p=0.76	p=0.46

The DBS OFF connectivity profile of optimal responders – classification analysis, details

The nested leave-one-out cross-validation design was adapted. In the outer loop, one subject was removed as a test subject, and the rest went into the inner loop. There, another subject was eliminated. On the remaining validation dataset, a sweep across different levels of response threshold for identifying optimal responders was undertaken. For each response threshold level, a subset of subjects was identified as possibly-optimal, and the average difference in DBS ON and DBS OFF connectivity was computed for this subset. The values of connectivity differences outside four standard deviations from the average value in each frequency band were used to define a characteristic connectivity profile of these possibly-optimal responders. This process is depicted in the scheme in Supplementary Figure S5.

The identified profile was used as an input to the linear support vector machine (SVM) classification to classify optimal and suboptimal responders. The procedure includes the following steps. Subjects identified as possibly-optimal were observed as positives (optimal responders) and the rest as negatives (suboptimal responders). Their individual connectivity profiles were taken to train a linear SVM classifier. The classifier was then evaluated on the test subject. Bringing the classification results across all folds together, correctly classified possibly-optimal responders were marked as true positives (TP) and correctly classified suboptimal subjects as true negatives (TN). The possibly-optimal responders incorrectly classified as suboptimal were marked as false negatives (FN), and suboptimal responders incorrectly classified as optimal were saved as false positives (FP). From these, the positive predictive value (PPV) was evaluated as PPV=TP/(TP+FP), negative predictive value (NPV) as NPV=TN/(FN+TN), sensitivity=TP/(TP+FN), and specificity=TN/(FP+TN).

The PPV, NPV, sensitivity, and specificity were used to evaluate the strength of the connectivity profile to predict optimal responders. When the response threshold is low, and a group of optimal responders contains all or almost all subjects (THR>-14), high sensitivity and high positive predictive values are obtained. However, the specificity and negative predictive values are low because suboptimal responders were included in the connectivity profile computation. At the other end of the spectrum, when only subjects with the best movement improvement after DBS were used in the analysis, it resulted in high specificity and negative predictive values, as well as very low sensitivity and positive predictive values. However, when the connectivity from a broader scale of subjects is included in connectivity profile computation, the percentage of correctly classified true optimal responders increases while keeping the positive predictive value relatively high.

In this way, a classifier was constructed for each response threshold of possibly-optimal responders.

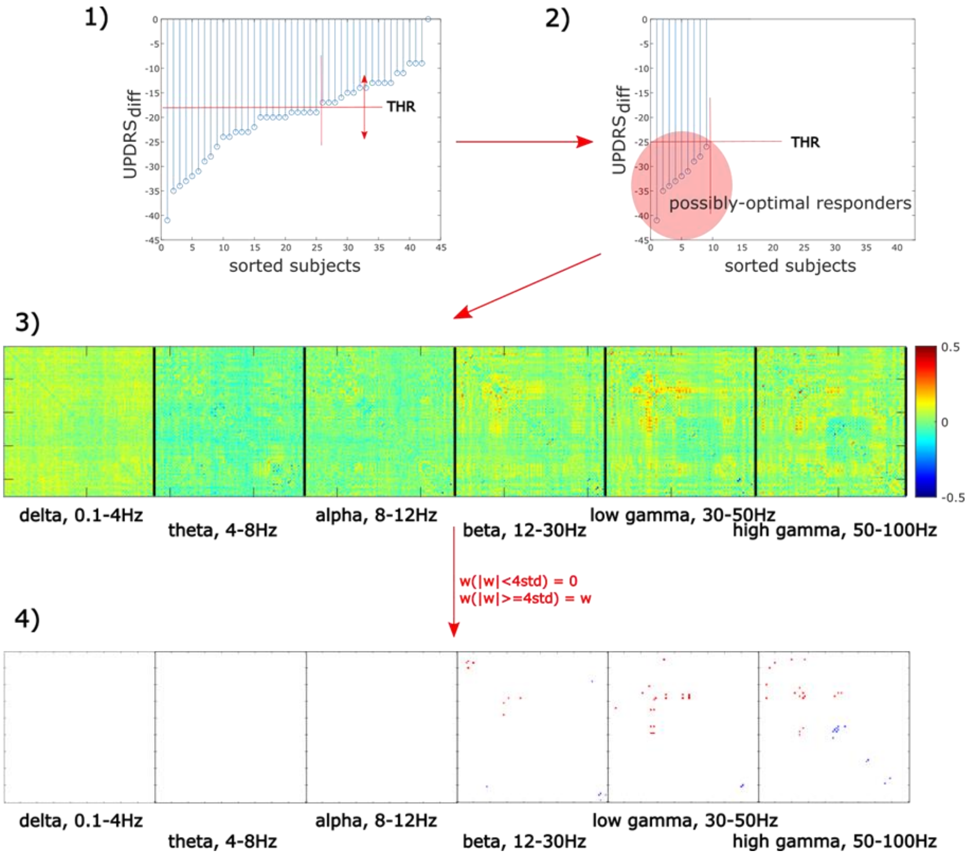

Figure S5: Scheme for extraction of connectivity profile from optimal responders. **1)** $UPDRS_{diff}$ = $UPDRS_{ON}$ – $UPDRS_{OFF}$ computed for each subject and sorted. Sweep across response thresholds identifying possibly-optimal responders ($UPDRS_{diff}$ from -9 (poor improvement of motor symptoms after DBS) to -41 (the best-measured improvement)). **2)** Set a response threshold for possibly-optimal responders. **3)** For them, compute subject-wise DBS ON – DBS OFF connectivity difference in each frequency band separately and compute the average across subjects. **4)** Use only extreme values of connectivity difference to specify the connectivity profile influenced the most by DBS (values outside four standard deviations from the average value in the matrix). Obtain the profile for each frequency band. Repeat the process from step 2) with a different response threshold.

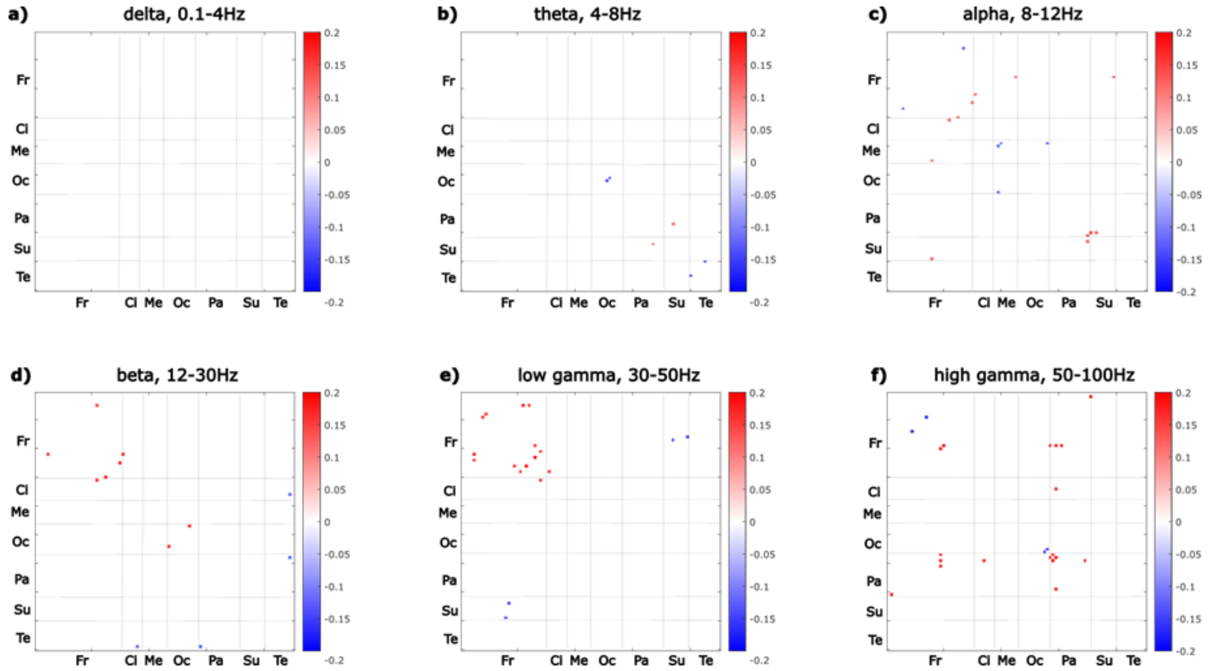

Figure S6: Matrix representation of the connectivity profile discriminating optimal and suboptimal responders the best. Red: increase in ON, blue: decrease in ON. Fr: Frontal, Ci: Cingulum, Me: Mesiotemporal, Oc: Occipital, Pa: Parietal, Su: Subcortical, Te: Temporal regions.

Table S4: Full names of ROIs involved in the connectivity profile discriminating response to DBS. ROIs are sorted. The first reported are those involved in increased connections in DBS ON, and the second are those involved in connections decreased in DBS ON compared to DBS OFF.

delta		theta		alpha	
		PCUN.L	Precuneus L	ORBinf.R	Frontal Inf Orb R
		PUT.R	Putamen R	OLF.R	Olfactory R
				ORBsupmed.L	Frontal Med Orb L
		MOG.L	Occipital Mid L	INS.R	Insula R
		MOG.R	Occipital Mid R	ACG.L	Cingulum Ant L
		HES.R	Heschl R	CUN.L	Cuneus L
		MTG.L	Temporal Mid L	PCL.R	Paracentral Lobule R
				CAU.L	Caudate L
				PUT.L	Putamen L
				HES.L	Heschl L
				ORBsup.R	Frontal Sup Orb R
				REC.L	Rectus L
				PHG.L	ParaHippocampal L
				PHG.R	ParaHippocampal R
				FFG.R	Fusiform R

beta		low gamma		high gamma	
ORBsup.L	Frontal Sup Orb L	ORBsup.L	Frontal Sup Orb L	PreCG.R	Precentral R
OLF.R	Olfactory R	MFG.R	Frontal Mid R	SMA.L	Supp Motor Area L
ORBsupmed.L	Frontal Med Orb L	ORBmid.L	Frontal Mid Orb L	SMA.R	Supp Motor Area R
INS.R	Insula R	SMA.L	Supp Motor Area L	DCG.R	Cingulum Mid R
ACG.L	Cingulum Ant L	OLF.L	Olfactory L	PoCG.L	Postcentral L
LING.L	Lingual L	OLF.R	Olfactory R	PoCG.R	Postcentral R
IOG.R	Occipital Inf R	SFGmed.L	Frontal Sup Medial L	SPG.L	Parietal Sup L
		SFGmed.R	Frontal Sup Medial R	IPL.L	Parietal Inf L

PCG.R	Cingulum Post R	ORBsupmed.R	Frontal Med Orb R	PCL.L	Paracentral Lobule L
PoCG.R	Postcentral R	REC.R	Rectus R	CAU.L	Caudate L
ITG.L	Temporal Inf L	ACG.L	Cingulum Ant L		
				ORBmid.L	Frontal Mid Orb L
		ORBinf.R	Frontal Inf Orb R	IFGtriang.R	Frontal Inf Tri R
		ROL.L	Rolandic Oper L	FFG.L	Fusiform L
		PUT.R	Putamen R	FFG.R	Fusiform R
		HES.L	Heschl L		

Table S5: Results of one repetition of the cross-validation. The PPV, NPV, specificity (Spec), and sensitivity (Sens) of each fold of the outer loop for the best response threshold (THR) identified by the inner loop.

fold	THR	PPV	NPV	Spec	Sens	fold	THR	PPV	NPV	Spec	Sens
**1**	17	0.89	0.55	0.86	0.63	**22**	18	0.79	0.55	0.75	0.60
**2**	18	0.82	0.68	0.76	0.75	**23**	23	0.80	0.75	0.96	0.31
**3**	18	0.74	0.50	0.69	0.56	**24**	18	0.76	0.55	0.69	0.64
**4**	16	0.82	0.54	0.58	0.79	**25**	18	0.81	0.56	0.82	0.54
**5**	17	0.78	0.50	0.64	0.67	**26**	18	0.75	0.52	0.76	0.50
**6**	18	0.76	0.55	0.69	0.64	**27**	18	0.74	0.55	0.71	0.58
**7**	16	0.76	0.40	0.62	0.57	**28**	16	0.79	0.50	0.50	0.79
**8**	18	0.72	0.48	0.69	0.52	**29**	18	0.79	0.59	0.76	0.63
**9**	18	0.76	0.55	0.69	0.64	**30**	16	0.77	0.47	0.54	0.71
**10**	18	0.76	0.54	0.76	0.54	**31**	16	0.78	0.50	0.54	0.75
**11**	18	0.67	0.48	0.65	0.50	**32**	16	0.81	0.60	0.50	0.86
**12**	18	0.71	0.50	0.71	0.50	**33**	18	0.75	0.52	0.69	0.60
**13**	18	0.83	0.67	0.75	0.76	**34**	16	0.86	0.62	0.67	0.83
**14**	18	0.79	0.52	0.82	0.46	**35**	18	0.77	0.58	0.69	0.68
**15**	17	0.92	0.45	0.93	0.41	**36**	21	0.75	0.76	0.93	0.43
**16**	18	0.75	0.52	0.69	0.60	**37**	18	0.80	0.62	0.76	0.67
**17**	23	0.67	0.74	0.93	0.31	**38**	20	0.62	0.61	0.77	0.42
**18**	18	0.76	0.55	0.69	0.64	**39**	17	0.74	0.44	0.57	0.63
**19**	24	0.75	0.81	0.97	0.30	**40**	16	0.77	0.47	0.54	0.71
**20**	18	0.81	0.56	0.82	0.54	**41**	16	0.82	0.54	0.58	0.79
**21**	17	0.74	0.41	0.64	0.52	**42**	18	0.75	0.57	0.71	0.63

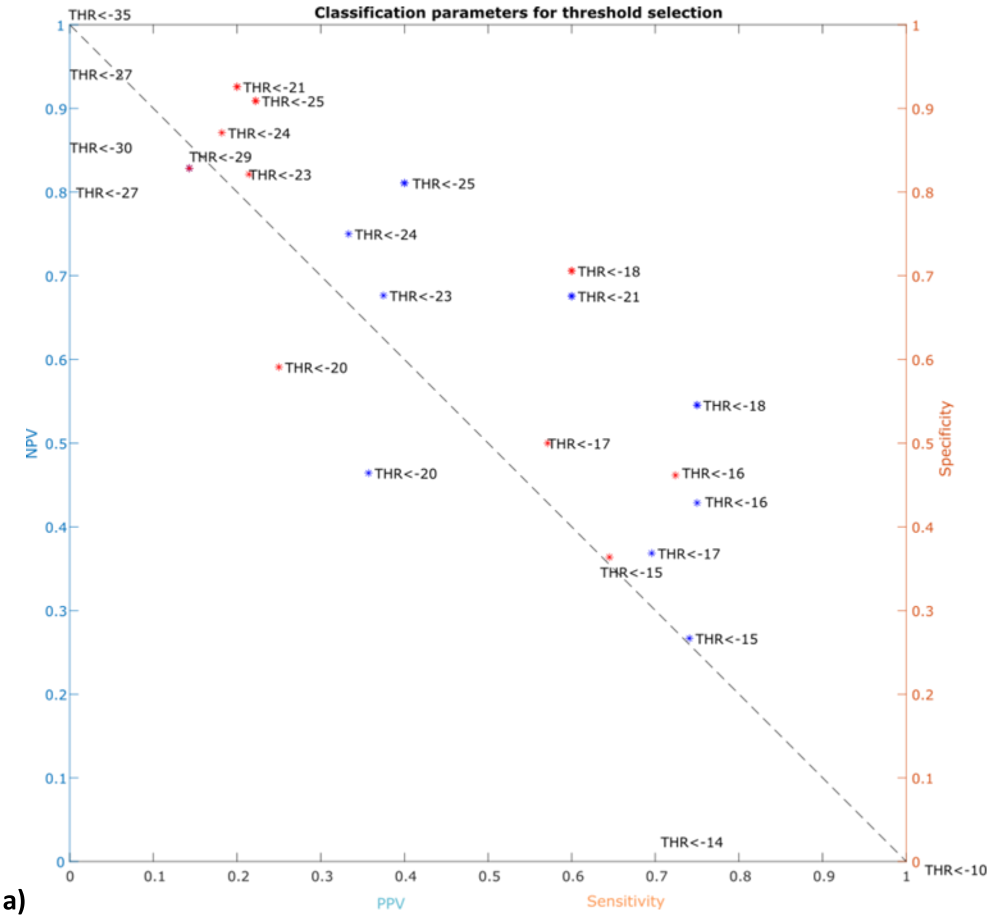

a)

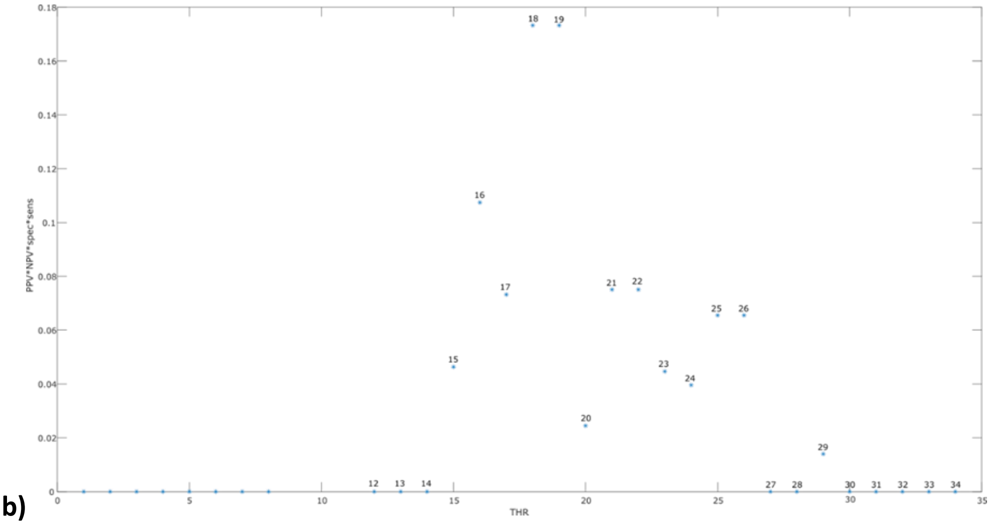

b)

Figure S7: a) The relations between positive predictive values (PPV) and negative predictive values (NPV), sensitivity, and specificity for diverse response thresholds for the 1st fold of the cross-validation outer loop. Blue points: Relation between NPV and PPV, red points: relation between specificity and sensitivity. **b)** Multiplication between PPV, NPV, spec, and sensitivity for diverse response thresholds. The highest y-value implies the best ratio of all four measures.

Rationale for temporal window selection (200 – 600 ms after stimulus onset) in PLI calculation

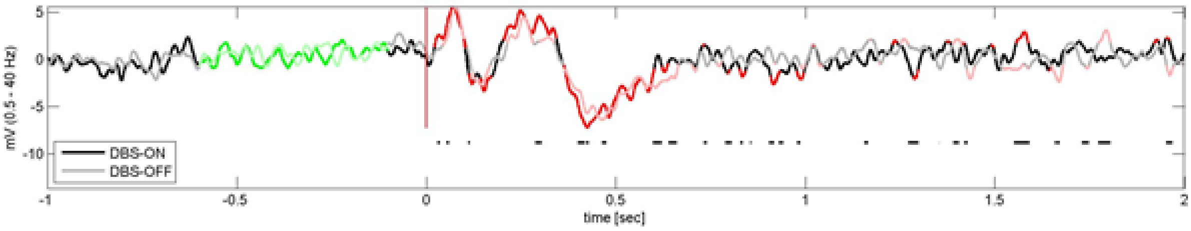

Figure S8: Example of ERP in SMA on target stimulus. The green color represents the baseline interval. The significant response is marked by red, and the significant difference between DBS ON and OFF conditions is shown by the dashed line under the time series.

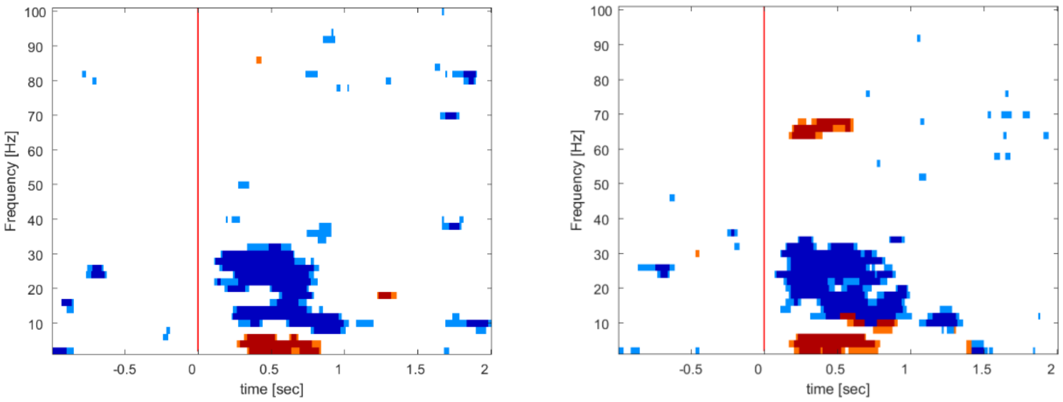

Figure S9: Example of time-frequency analysis in SMA after target stimulus onset. Blue corresponds to event-related desynchronization (significant power decrease), red indicates event-related synchronization (significant power increase). DBS OFF on the left, DBS ON condition on the right.
